# Current strategies for the design of PROTAC linkers: a critical review

**DOI:** 10.37349/etat.2020.00018

**Published:** 2020-10-30

**Authors:** Robert I. Troup, Charlene Fallan, Matthias G. J. Baud

**Affiliations:** 1 School of Chemistry, University of Southampton, Highfield, SO17 1BJ Southampton, UK; 2 Medicinal Chemistry, Oncology R&D, AstraZeneca, Cambridge Science Park, Milton Road, CB4 0WG Cambridge, UK; Istituto Nazionale Tumori “Fondazione Pascale” Via Mariano Semmola, Italy

**Keywords:** PROTAC, protein degradation, linker design

## Abstract

PROteolysis TArgeting Chimeras (PROTACs) are heterobifunctional molecules consisting of two ligands; an “anchor” to bind to an E3 ubiquitin ligase and a “warhead” to bind to a protein of interest, connected by a chemical linker. Targeted protein degradation by PROTACs has emerged as a new modality for the knock down of a range of proteins, with the first agents now reaching clinical evaluation. It has become increasingly clear that the length and composition of the linker play critical roles on the physicochemical properties and bioactivity of PROTACs. While linker design has historically received limited attention, the PROTAC field is evolving rapidly and currently undergoing an important shift from synthetically tractable alkyl and polyethylene glycol to more sophisticated functional linkers. This promises to unlock a wealth of novel PROTAC agents with enhanced bioactivity for therapeutic intervention. Here, the authors provide a timely overview of the diverse linker classes in the published literature, along with their underlying design principles and overall influence on the properties and bioactivity of the associated PROTACs. Finally, the authors provide a critical analysis of current strategies for PROTAC assembly. The authors highlight important limitations associated with the traditional “trial and error” approach around linker design and selection, and suggest potential future avenues to further inform rational linker design and accelerate the identification of optimised PROTACs. In particular, the authors believe that advances in computational and structural methods will play an essential role to gain a better understanding of the structure and dynamics of PROTAC ternary complexes, and will be essential to address the current gaps in knowledge associated with PROTAC design.

## Introduction

### General considerations

**Figure 1.**
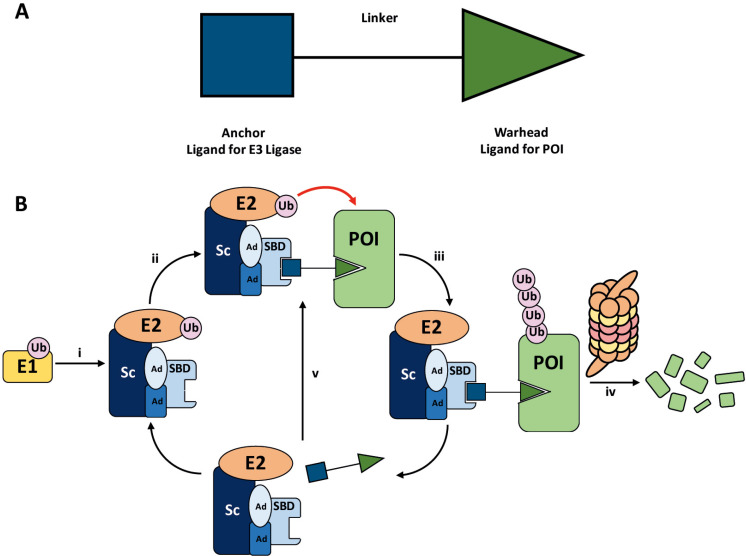
A. General structure of a PROTAC. The E3 ligase targeting “anchor” (blue) is connected to the specific POI targeting warhead (green) via a variable linker; B. mechanism of PROTAC-mediated target degradation via RING-type E3 ligases. (i) Ub transfer from E1 to E2 by trans-thioesterification is followed by complex formation with an E3 ligase; (ii) the PROTAC binds to both the E3 ligase and POI to form a TC, where the E3 ligase is shown as an assembly of scaffolding proteins (Sc), adapter proteins (Ad), and a SBD. This brings the E2 ligase into proximity to the POI; (iii) this leads to the transfer of multiple Ub units to surface exposed lysine residues; (iv) the resulting polyubiquitin chain is recognised by the proteasome, leading to the proteolytic degradation of the POI; and (v) the PROTAC is released and can catalyse the transfer of Ub to additional POIs

Proteolysis targeting chimeras (PROTACs) are heterobifunctional molecules consisting of two ligands connected by a linker [1–5]. An “anchor” ligand binds to the substrate binding domain (SBD) of an E3 ubiquitin (Ub) ligase, and a “warhead” ligand binds to a particular protein of interest (POI) to be targeted (Figure 1A). Through binding to both proteins in cells, the PROTAC recruits the POI to a ternary complex (TC) with the E3 ligase [6]. The E3 ligase itself is in complex with an activated Ub-loaded E2 ligase, and the TC formation brings the ensemble into close proximity with the POI. This induces the (poly)-ubiquitination of the POI at lysine residues, marking it for degradation by the 26S proteasome (Figure 1B) [3, 7, 8].

PROTACs act as adapter molecules between the E3 ligase and any chosen POI, hijacking the activity of the cell’s natural protein degradation machinery, i.e. the ubiquitin-proteasome system (UPS). A significant proportion of E3 ligases are multiprotein complexes and are usually composed of a Sc and SBD, bound via Ad. It is estimated that the human proteome contains > 600 E3 ligases, whose exquisite substrate specificities are guided by their individual molecular architecture resulting from distinctive combinations of Sc, Ub-loaded E2, Ad, and ultimately SBD [8–10].

**Figure 2.**
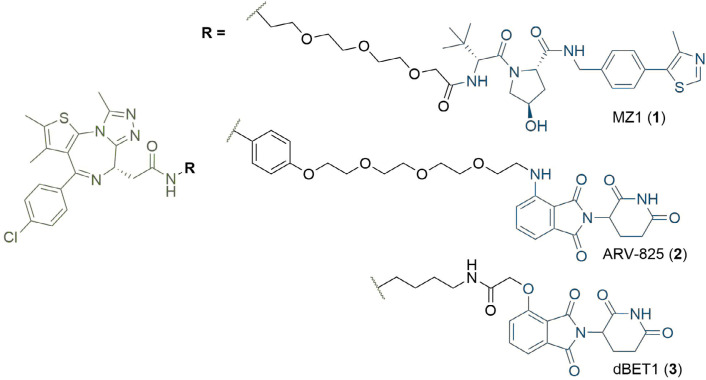
Structures of BRD4 degraders MZ1 (1), ARV-825 (2), and dBET1 (3). The anchors of (1) and (2–3) targeting the Von Hippel-Lindau tumour suppressor protein (VHL) and cereblon (CRBN) respectively are highlighted in blue; the common JQ1 based warhead is highlighted in green, and the linkers in black

The degradative mechanism of action of PROTACs sits in stark contrast to traditional small-molecule inhibitors, which typically antagonise targets through binding to a functional or allosteric site, and this presents several notable advantages. Instead of an occupancy driven effect, PROTACs exert their inhibitory effects via “event-driven” pharmacology. This mechanism is catalytic, and PROTAC molecules freed from the TC can elicit degradation of multiple POIs [11]. Crucially, the high catalytic turnover and irreversible action of the UPS allows PROTACs to be used at extremely low concentrations (down to pM) in cells, which represents a major advantage compared to “occupancy-based” inhibitors [12]. Another key feature of PROTACs is that the binding site/mode of the warhead to the POI is not of primary importance for successful ubiquitination and does not necessarily need to be functional, as long as the warhead provides sufficient affinity to recruit the POI to the complex. This could provide a means to target the estimated 80% of the human proteome thought to be intractable to conventional small-molecule methods [such as protein-protein interactions (PPIs) and Sc] due to their lack of a well-defined functional binding site, as found in enzymes, G-protein-coupled receptors, and ion channels [13]. For example, PROTACs have been designed to degrade the transcription factor STAT3, despite it having proved obstinate to traditional small molecule therapeutics [14]. Finally, the formation of a TC provides an opportunity to impart an additional layer of affinity and selectivity beyond that of the formation of a binary PROTAC-POI complex, since additional PPIs or protein-PROTAC interactions can favour its formation and stability. The Bromo- and Extra-Terminal (BET) bromodomain degrader MZ1, developed by Zengerle et al. [12], provides a particularly stark example of this (1, Figure 2). The molecule uses the potent pan-selective BET inhibitor JQ1 as a warhead, but the PROTAC is relatively selective for BRD4 degradation over BRD2 or BRD3 in HeLa cells. Contrastingly, the highly potent BRD4 degraders ARV-825 (2) and dBET1 (3) utilise a different E3 ligase and differ in linker structures, and are also able to degrade BRD2 and BRD3 very efficiently [15, 16].

**Figure 3.**
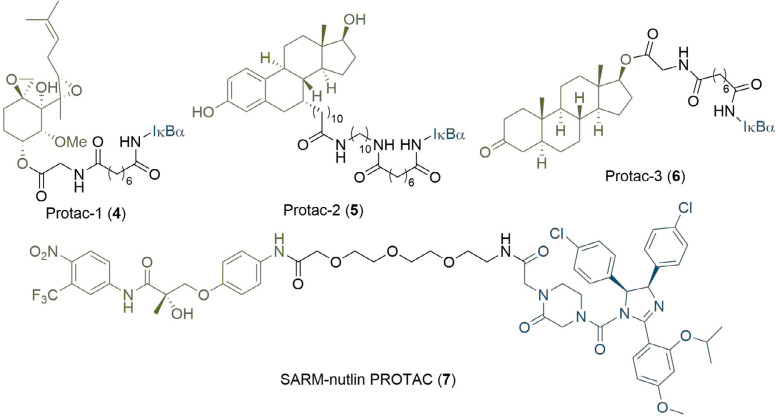
Early PROTAC development: the first published PROTAC degrader (4), which conjugates the angiogenesis inhibitor ovalicin to the IκBα phosphopeptide (denoted IκBα); structures of second generation PROTACs targeting the ER (5) and AR (6) receptors; the first all small-molecule PROTAC incorporating the MDM2 ligand nutlin-3 (7). In all PROTACs, the anchor is coloured blue and the warhead green

PROTAC research to date has predominantly concerned the development of potent E3 binding ligands and expanding the methodology to new POI targets. The first PROTAC reported (4, Figure 3), in seminal work by Sakamoto et al. [17], consisted of a ligand (ovalicin derivative) for mammalian methionine aminopeptidase type 2 (MetAP-2) connected by a flexible alkyl linker to an IκBα phosphopeptide, which is recognised by the Skp1-Cullin-F box complex (SCF). Protac-1 (4) was able to artificially recruit MetAP-2 to SCF^β-TRCP^ for polyubiquitination and subsequent proteasomal degradation. In a follow-up study, the general applicability of the strategy was further demonstrated through the development of PROTAC derivatives of oestradiol (5) and dihydroxytestosterone (6, Figure 3) to degrade the androgen (AR) and oestrogen (ER) receptors respectively [18]. However, the highly polar peptidic ligase-binding sequences of these early PROTACs caused poor cell permeability, presented potential issues with their proteolytic stability, and consequently limited their therapeutic scope [19]. This provided impetus for the development of non-peptidic small-molecule PROTACs, the first of which was reported by Schneekloth et al. [20], in 2008. PROTAC 7, containing a nutlin-3 anchor, displayed enhanced cell permeability and could target the AR for degradation via murine double minute 2 (MDM2), although micromolar concentrations of the compound were required to elicit measurable degradation. Further refinements have been made using MDM2: Hines et al. [21], later developed PROTACs with nanomolar potencies, which inhibited proliferation of several cancer cells lines through a synergistic effect of simultaneous BRD4 degradation and p53 stabilization.

### PROTAC anchors

**Figure 4.**
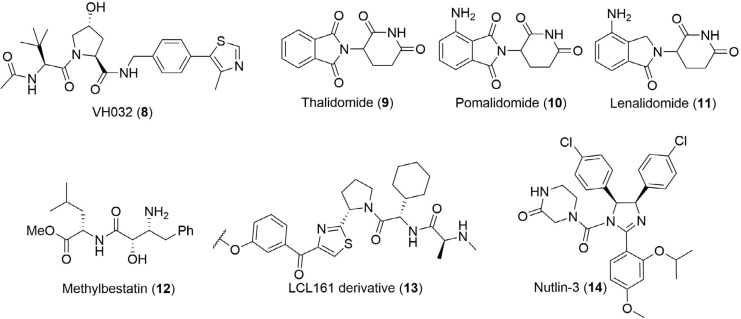
Commonly used anchor ligands. Structure of the high-affinity ligand VH032 (8) commonly used to recruit VHL. Structures of thalidomide (9) and its analogues pomalidomide (10) and lenalidomide (11), which recruit CRBN. Methylbestatin (12) and a higher-affinity derivative of LCL161 (13) are most commonly used to target cIAP. Nutlin-3 (14) has been used to target MDM2

More recently, E3 cullin-RING ligases have attracted significant attention for ligand development [22]. Substantial efforts by the Crews and Ciulli labs have been devoted to the development of potent small molecules binding to VHL [23, 24] and disrupting its interaction with the α-subunits of the hypoxia-inducible factor 1α (HIF-1α), a key regulator of the cellular hypoxic response [25, 26]. Ligands targeting this PPI were initially developed as potential drug candidates for the treatment of ischemic disease [27] and subsequently exploited for the development of PROTACs. Representative hydroxyproline based molecule 8 (Figure 4) is among the most potent VHL ligands (*K_d_* = 185 nM) [23], and was employed for the development of MZ1 (1) in 2015 [12]. VHL-based degraders of the protein kinase RIPK2 and the orphan nuclear receptor ERRα with nanomolar cellular potencies were also reported around this time [11]. The concurrent discovery of thalidomide (9) and its analogues (10, 11) as ligands for the E3 ligase CRBN enabled the development of ARV-825 (2) and d-BET1 (3) as potent BET bromodomain degraders [14, 15]. Of note, this also highlighted that the activity of PROTAC molecules is not limited to the cytoplasm, but can induce potent protein knock-down in the nucleus [12]. Despite the discovery of high-affinity ligands for MDM2, VHL and CRBN, expanding the scope of E3 ligase ligands has proven more challenging. Over 600 E3 ligases have been identified in the human proteome [8, 28], but there is a general dearth of high affinity ligands available for them [29]. However, the option to target different ligases is important: changing the recruited ligase has been shown to alter the degradation profile of PROTACs [30]; their expression can vary amongst different cell lines; and mutations in the core components of E3 ligase complexes can result in cells acquiring resistance to PROTAC action [31]. In addition to VHL and CRBN, PROTAC-like molecules targeting cellular inhibitor of apoptosis protein (cIAP) have also been widely reported [32]. These are more commonly referred to as specific and nongenetic IAP-dependent protein erasers (SNIPERs) [33]. Methyl bestatin (12) was initially used as the anchor [34], but a higher affinity ligand (13) based on LCL161 has since been developed [35, 36]. Examples of PROTACs utilizing CUL4-DDB1 [37], RNF4 [38], and Keap1 [39] have also been reported. Ottis et al. [40], took a unique approach to identifying novel E3 ligase targets, and instead looked at engineering their SBDs to accept a particular ligand. Of the six ligases they modified, five were able to recruit proteins for targeted degradation.

### PROTAC warheads

**Figure 5.**
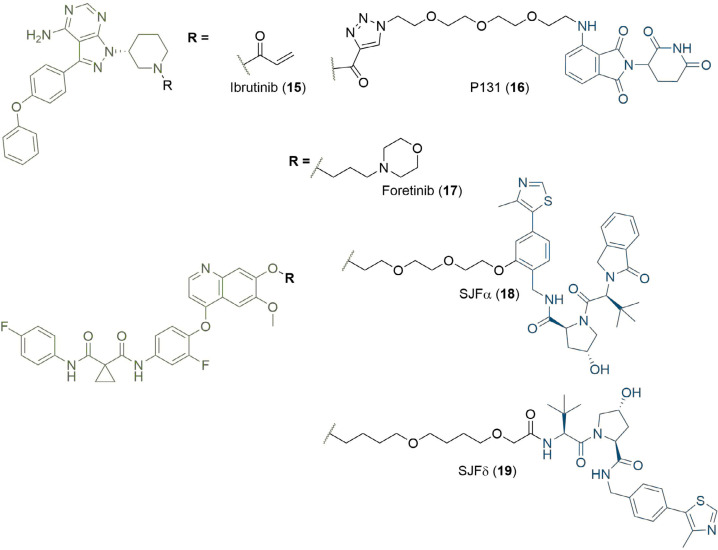
Structure of the BTK covalent inhibitor ibrutinib (15) and its PROTAC counterpart P131 (16), a potent degrader of WT and C481S mutant BTK. Promiscuous kinase inhibitor foretinib (17) is incorporated into PROTAC degraders of MAPK kinases, which differ by linker composition and site of conjugation to the VHL ligand. SJFα (18) is selective for p38α and SJFδ (19) for p38δ. In all PROTACs, the anchor is coloured blue and the warhead green

The warhead ligand can be readily substituted to develop degraders for myriad POIs, and this has enabled the rapid expansion of the modality to target proteins implicated in many diseases; degraders have now been reported against over 40 different cellular protein targets [2]. A significant proportion of these proteins (> 80%) are implicated in various types of cancers, although other disease states are also represented. Examples include: PROTACs targeting interleukin-1 receptor-associated kinase 4 (IRAK4) for the treatment of autoimmune and inflammatory disease [41]; PROTAC degraders of viral proteins for inhibition of the hepatitis C virus [42]; and Tau degraders for the treatment of Alzheimer’s disease [43, 44]. Notable cancer POIs targeted by PROTAC degraders include transcription factors such as the aryl hydrocarbon receptor [45]; kinases such as the BCR-ABL fusion protein [30]; dual degraders of Cyclin-dependent kinases 4 and 6 (CDK4/6) [46]; EGFR [47]; and a range of important epigenetic effectors from the bromodomain and histone deacetylase (HDAC) families [48, 49]. The PROTAC modality can be useful against targets that are intractable to traditional small molecule therapeutics, such as the Cys481 to Ser (C481S) mutant Bruton’s tyrosine kinase (BTK). Inhibition of BTK activity is an established strategy for the treatment of Non-Hodgkin’s lymphoma and chronic lymphocytic leukaemia, which arise from B-cell malignancies [50]. Important to the activity of the first-in-class covalent BTK inhibitor ibrutinib (15, Figure 5) is its acrylamide unit, acting as a mild Michael acceptor and leading to irreversible covalent binding with Cys481 at the entrance of the BTK active site. As expected, ibrutinib activity is highly susceptible to the C481S mutation due to this loss of covalent binding; a 74-fold potency decrease has been observed between mutant and wild-type BTK [51]. PROTACs are emerging as a promising alternative to circumvent the resulting ibrutinib resistance, with many examples now reported [52]. For example, PROTAC 16 (Figure 5), developed by Sun et al. [53], was able to degrade both wild-type and C481S mutant BTK with nanomolar potencies. Another advantage is that PROTACs can permit isoform selective degradation across families of proteins sharing high sequence and structural homology in their binding sites, but which present significant structural diversity at their surface. The BET proteins represent a particularly compelling example of this. All eight BET bromodomains share a high degree of structural homology which makes developing selective inhibitors challenging. Gadd et al. [54], were able to induce stabilising interactions in the TC via linker optimisation to achieve BRD4 selectivity. Another example of this is shown in foretinib-based degraders of the MAPK family developed by the Crews group. The c-Met tyrosine kinase inhibitor foretinib (17) is highly promiscuous: it binds to 133 different kinases with high affinity [55]. However, when 17 was conjugated to a derivative of VH032 (8) by Smith et al. [56], differential substrate selectivity could be obtained based on the length, composition, and attachment point of the linker. By varying these parameters, selective degraders of either p38α (SJFα, 18) or p38δ (SJFδ, 19) could be obtained (Figure 5). The development of the PROTAC technology for the treatment of cancer has ultimately culminated in the first two degraders (both from Arvinas, undisclosed structures) recently reaching phase 1 clinical trials, where their safety and tolerability are being assessed through dose-escalation [57]. ARV-110 is being tested in patients with metastatic castration-resistant prostate cancer, and ARV-471 in patients with ER+/human epidermal growth factor receptor 2 (HER2)-locally advanced or metastatic breast cancer [58].

### PROTAC linkers

In contrast to the wealth of literature concerning modification of the two protein-binding ligands, reports focused on the linker specifically are less common. It has become evident that the overall degradation efficiency does not simply rely on the affinities of the anchor/warhead for the E3 and POI respectively, but rather on the judicious combination of anchor and warhead connected by a suitable linker, allowing productive TC formation and POI ubiquitination [2, 3]. Indeed, it is now well supported that the length and composition of the linker is very important for productive TC formation, degradation activity, and target selectivity. A number of recent studies have pointed at the important role of the linker for positive cooperative TC assembly, where the linker engages in specific interactions in the TC [54, 59]. These observations have potentially profound implications for the design of PROTACs displaying isoform selectivity across families of structurally related POIs [60]. Equally important, linker mediated binding cooperativity also represents a potential source of affinity for the POI for PROTACs based on weak affinity warheads [59]. However, the current consensus is that the linker composition, and particularly its length and attachment point to the anchor/warhead, must be optimised for each ligand pair. This is not surprising, as the structural complexity and dynamics of the TC make it a formidable challenge to predict which combination of anchor/linker/warhead will lead to optimal degradation. As a result there is currently no generally applicable strategy for linker design; bioactivity optimisation through synthetic alteration of the linker is usually achieved via iterative trial and error, often using short and structurally simple alkyl or PEG chains as starting points. Beyond degradation, linkers have been exploited to encode new chemical functionalities into the PROTAC; this include a range of photoswitches, conformational locks, and covalent warheads. Despite their critical importance, to our knowledge there are no comprehensive reviews on PROTAC linker chemistry and design strategy (although Borsari et al. [61], provide a general overview of linker chemistry in chimeric molecules). In the next section, we will review and summarise the diverse chemical motifs that have appeared in published degrader structures, and discuss the associated design approaches taken towards optimising their linker unit.

## Current elements of PROTAC linker design

There are currently no generally accepted rules for *de novo* PROTAC linker design that can ensure the generation of a potent degrader for any given E3-POI pair, and some degree of empirical trial and error is often required. However, historically most PROTAC linkers have consisted of combinations of only a few main chemical motifs. This was recently highlighted by Maple et al. [62], who compiled a database of over 400 published degrader structures. Some of their findings concerning the prevalence of different linker motifs are summarised in Table 1. By far the most common motifs incorporated into PROTAC linker structures are PEG and alkyl chains of varying lengths, and these are the sole motif in approximately 55% and 30% of linkers respectively. Around 65% of structures in the database contained both an alkyl and PEG segment. A further 15% used modifications of the individual glycol units, incorporating additional methylene moieties to access different chain lengths. Other represented motifs include alkynes (7%), triazoles (6%) and saturated heterocycles such as piperazine and piperidine (4% each).

**Table 1.** Occurrence of selected linker motifs in the Maple database of published degrader structures. Wavy lines indicate attachment to other linker motifs, protein-binding ligands, or connecting functional groups. Since many PROTACs combine more than one structural motifs into their linkers therefore these percentages sum to more than 100

**Structure**	**Linker motif**	**Occurrence in Maple Database structures (%)**
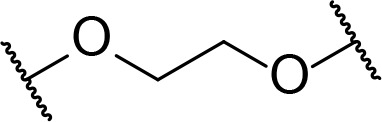	PEG	54
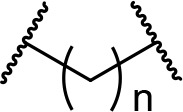	Alkyl	31
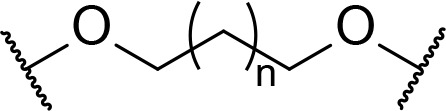	Other Glycol	14
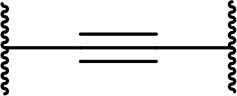	Alkyne	7
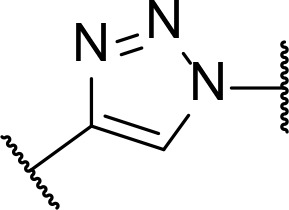	Triazole	6
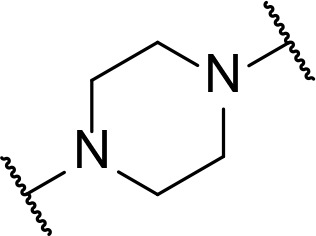	Piperazine	4
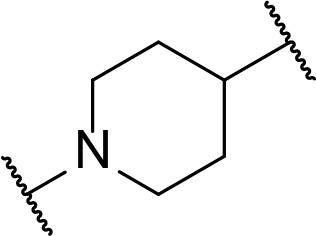	Piperidine	4

### Alkyl and PEG chains

Alkyl, PEG, and extended glycol chains are by far the most common linker motifs appearing in published degrader structures, and there are some key advantages to these compositions that underlie their prevalence in the literature. These include their synthetic accessibility, their flexibility, and the ability to easily tune their length and composition via a wide array of robust chemical methods. Using diverse combinations of PEG and alkyl motifs also allows for tuning of important physical properties such as topological polar surface area (TPSA) and lipophilicity. These in turn have implications for properties like solubility and cell permeability that affect oral absorption, and will be discussed later.

The modular nature of PROTACs can enable their rapid assembly from the sequential conjugation of the warhead and anchor (or vice versa) ligands to the linker. For this purpose, it is convenient to have a pre-assembled linker molecule that can be efficiently functionalised at either end through orthogonal conditions or deprotection sequences. The commercial availability of such bi-functionalised alkyl and PEG motifs enables the rapid and facile assembly of potent degrader structures when these are used as linkers [63]. A range of such linker motifs pre-conjugated to E3 ligands such as pomalidomide can also be obtained from commercial sources [64].

**Figure 6.**
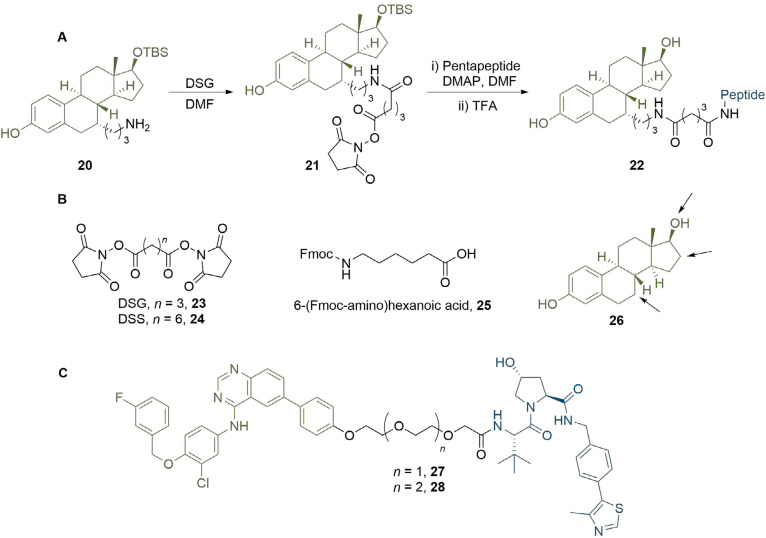
Effect of PROTAC linker length and conjugation site. A. In a representative PROTAC synthesis by Cyrus et al. [65], 20 was reacted with DSG to install an amide-linked alkyl linker. The product 21 was then reacted with the E3-binding pentapeptide sequence to displace the second succinimidyl moiety and obtain PROTAC 22; B. commercially available linker building blocks used by Cyrus et al. [65], include DSG (23), DSS (24), and Fmoc-protected acid 25. The structure of oestradiol (26) is shown with arrows pointing to potential sites of linker conjugation; C. extension of the PEG linker in EGFR and HER2 degrader 27 by one unit abolished HER2 activity to afford selective EGFR PROTAC 28

The importance of linker length to degradation efficiency is well established, and alkyl or PEG motifs provide means to easily and systematically vary the length of the linker. In early work, Cyrus et al. [65], sought to develop a generalised approach to PROTAC synthesis through delineating this dependence. They synthesised ER degraders by conjugating oestradiol to a pentapeptide sequence derived from HIF-1α, which serves as the minimum recognition domain for VHL [66]. This sequence further contains a polyarginine tail to confer cell permeability [6]. Interestingly, potency increased as the linker length increased from 9 atoms [half maximal inhibitory concentration (IC_50_) = 140 μM)] to 16 atoms (IC_50_ = 26 μM), with the latter displaying similar cell viability reduction as the Tamoxifen control (IC_50_ = 27 μM) in MCF7 cancer cells. In contrast, compounds with longer linker lengths exhibited a sharp decrease in potency (IC_50_ > 200 μM) and clearly highlighted an optimal range of linker length, although the precise reasons were not investigated further. Their use of alkyl linkers enabled them to build up PROTACs from common amine intermediate 20 using commercially available building blocks such as di(*N*-succinimidyl) glutarate (DSG, 23), di(*N*-succinimidyl) suberate (DSS, 24) and 6-(Fmoc-amino)hexanoic acid (25) (Figure 6). The site of linker conjugation and its exit vector are also known to be important for degradation potency, which Cyrus et al. [67], explored in related work. They assessed the impact of different conjugation sites to oestradiol (26) on the activity of their PROTACs using DSS or DSG to introduce the alkyl fragments (Figure 6). Alterations in linker length can also be used to impart selectivity for degradation of different proteins [68]. For example, a lapatinib based PROTAC (27), developed by Burslem et al. [47], was able to degrade both EGFR and HER2 in OVCAR8 cell line. However, extension of the linker by a single ethylene glycol unit abolished HER2 degradation and provided a selective EGFR degrader (28).

**Figure 7.**
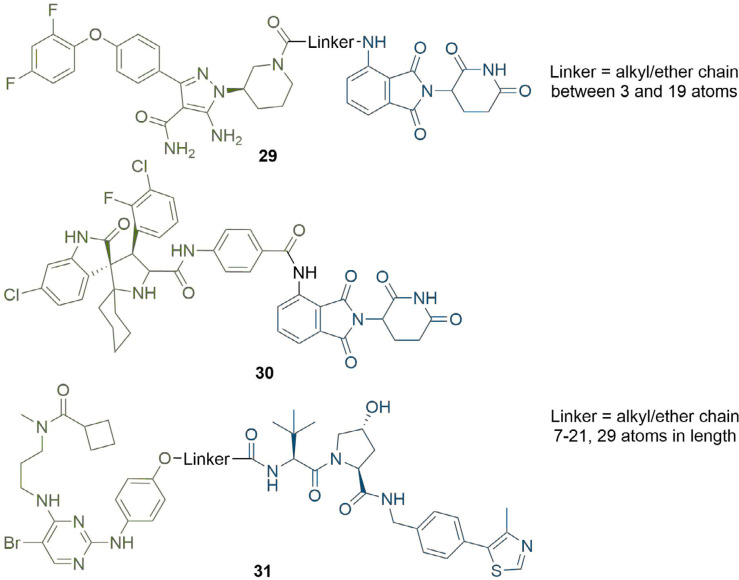
Structures of representative PROTACs with PEG/alkyl linkers. BTK degraders (29) where the linkers are alkyl/ether chains of various combinations between 3 and 19 atoms. MDM2-targeting PROTAC with direct conjugation of the warhead to the anchor (30), although this did not function as a degrader. TBK1 PROTACs with alkyl/ether linkers between 7 and 29 atoms in length (31). In all PROTACs, the anchor is coloured blue and the warhead green

When considering linker length, there is usually a minimum distance required between the warhead and anchor for a PROTAC to be effective. In a series of BTK degraders reported by Zorba et al. [60], binding affinity for BTK and CRBN was consistent between the free ligands and longer linker PROTACs (≥ 4PEG units), but was impaired by up to 20-fold for their shorter PROTACs (29, Figure 7). They rationalised this as being due to binary steric repulsions between one of the ligands and either of the proteins when bound to the other. Again, this is not a general rule, and potent PROTACs with linkers as short as three atoms have been reported [69]. Li et al. [70], even reported a PROTAC targeting MDM2 with the anchor and warhead directly connected without a linker (30). However, whilst this was able to potently inhibit cell growth in the RS4; 11 cell line (IC_50_ = 68 nM), MDM2 protein levels remained unaffected. In a related study by Yang et al. [71], the authors unexpectedly discovered that structural modifications of an MDM2 PROTAC degrader can result in “molecular glues”. The latter did not affect MDM2 levels, but rather induced potent cell growth inhibition by inducing degradation of the translation termination factor GSPT1.

When designing degraders incorporating new anchor/warhead pairs, the flexibility imparted by using long alkyl or ether chains can be crucial to find a potent compound. PROTACs targeting TBK1 were synthesised by Arvinas with linkers ranging from 7 to 29 atoms in length by various combinations of alkyl and ether units (31) [72]; below 12 atoms, TBK1 degradation was not observed. In contrast, compounds with linkers between 12 and 29 atoms all exhibited submicromolar degradation potency [half-maximal degradation concentration (DC_50_) = 3 nM and maximum degradation efficacy (D_max_) = 96% for the 21 atom linker], although with a decrease in potency at 29 atoms (DC_50_ = 292 nM and D_max_ = 76%). They hypothesised that the flexible nature of the linkers allowed them to adopt suitable conformations for productive TC formation at a range of lengths, but only once a minimum linker length was reached. This requirement is particularly evident in seminal work by the Ciulli group, who solved the crystal structure of degrader MZ1 (1) in complex with VHL and the second bromodomain (BD2) of BRD4 [protein database (PDB) 5T35] [54]. Interactions supporting the positive cooperativity of the TC formation are facilitated by the folding of the linker on itself to achieve its bioactive conformation, which necessitates a certain degree of flexibility. However, this is not always the case; Zorba et al. [60], observed that their BTK PROTACs (29) with longer linkers lacked positive cooperativity in the TC (although were still potent, DC_50_ 1-40 nM in Ramos cells). They rationalised this as being due to the energy gained in the TC from new PPIs being offset by the entropic cost of reduced PROTAC flexibility.

The atomic composition of the linker can also have significant effects on the potency of the PROTAC. Degraders generated by the conjugation of VHL and CRBN ligands with an alkyl linker were able to induce concentration dependent decrease of CRBN level in HEK293T cells [73]. However, exchange of a nine atom alkyl chain for three PEG units led to only weak CRBN degradation, which implied that the incorporation of oxygen in place of CH_2_ groups was somehow inhibiting the PROTAC activity, although the associated mechanism was not explored. In the aforementioned TC crystal structure of MZ1 (1), the ether oxygen atom adjacent to the amide bond to JQ1 makes a hydrogen bond interaction to a BD2-specific histidine (His437) [54]. This interaction would presumably be lost if the composition of the linker was altered from PEG to alkyl.

**Figure 8.**
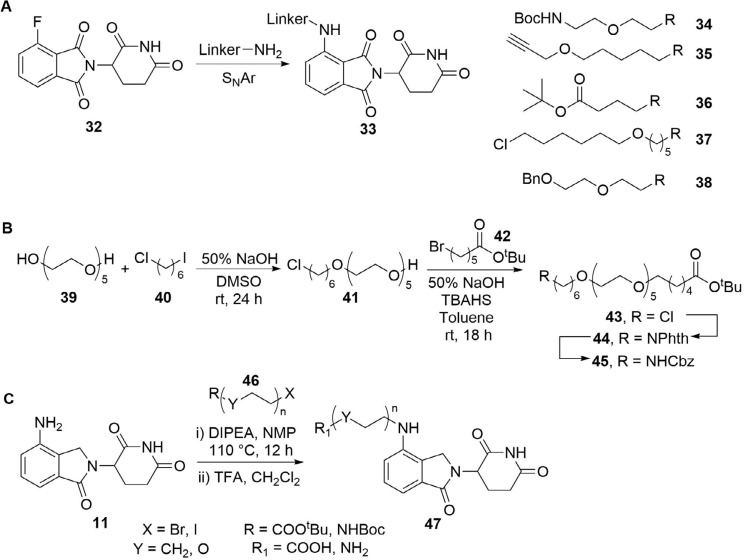
Key methods to assemble PROTAC libraries using alkyl and ether linkers. A. Nucleophilic aromatic substitution of 32 with linkers carrying an array of functionalities (34–38) was used to build a toolbox of compounds for CRBN PROTAC development (33); B. representative example synthesis of linkers with varying ether combinations; commercially available 39 was sequentially alkylated with 40 and 42. The chloride handle in 43 was subsequently converted to a Cbz-protected amine (45) after further manipulations; C. synthesis of a PROTAC library (47) by alkylation of lenalidomide (11) with various alkyl bromides/iodides (46)

The subtle effects of linker length and composition on degradation efficiency often create a requirement for significant empirical trial and error to produce an optimised linker structure. There has been an intense focus in the last decade on developing efficient and versatile synthetic methods to access diverse linker structures to enhance PROTACs bioactivity. For example, Steinebach et al. [74], devised a “toolbox” for the development of CRBN-directed PROTACs which contains a selection of mixed PEG and alkyl linkers with different C/O ratios so as to span a range of lengths and lipophilicities. Each linker was conjugated to pomalidomide (10) by the nucleophilic aromatic substitution (S_N_Ar) reaction of a primary amine with 4-fluorothalidomide (32). The other end of the linkers contains various functionalities to facilitate conjugation of a warhead by different routes, such as BOC-deprotection followed by amide coupling (34–38, Figure 8). Using this toolbox, a library of PROTACs with different linker lengths, compositions, and properties could be synthesised rapidly to probe structure-activity relationship (SAR). In separate work, Steinebach et al. [75], developed a series of linkers with orthogonally protected amine and acid termini. These linkers contained ethers with varying numbers of carbons between the oxygens in the repeating unit, which was conveniently indicated via a code (e.g., 2-2-2 indicates 2 carbons between each heteroatom in the chain), and were assembled from the sequential coupling of diverse alkyl halide building blocks. In a representative synthesis (Figure 8), diol (39) was mono *O*-alkylated with nonsymmetric dihaloalkane (40), then capped with bromide (42). The Gabriel synthesis was then used to install a phthalimide-protected nitrogen to afford 44, and a subsequent protecting group switch gave orthogonally protected linker 45 (coded 6-(2)_5_-6). In a complementary approach, Qiu et al. [76], devised fine-tuned conditions to chemoselectively alkylate the poorly nucleophilic aryl amine in lenalidomide (11), using alkyl bromides or iodides (46) and *N,N*-diisopropylethylamine (DIPEA) (Figure 8). These conditions provided synthetic access to a library of PEG and alkyl containing linkers of different lengths, bearing either a terminal amine or carboxylic acid handle for conjugation of the warhead (47). Many of these alkyl halide linkers could be conveniently obtained from commercial sources with no prior assembly required. Whilst this was successful when lenalidomide was the anchor, the additional carbonyl present in the more commonly employed pomalidomide (10) reduces the nucleophilicity of the aryl amine further, and *N*-alkylation is not frequently employed here: the aforementioned S_N_Ar with 32 is more common.

### Recent advances in PROTAC linkers

**Figure 9.**
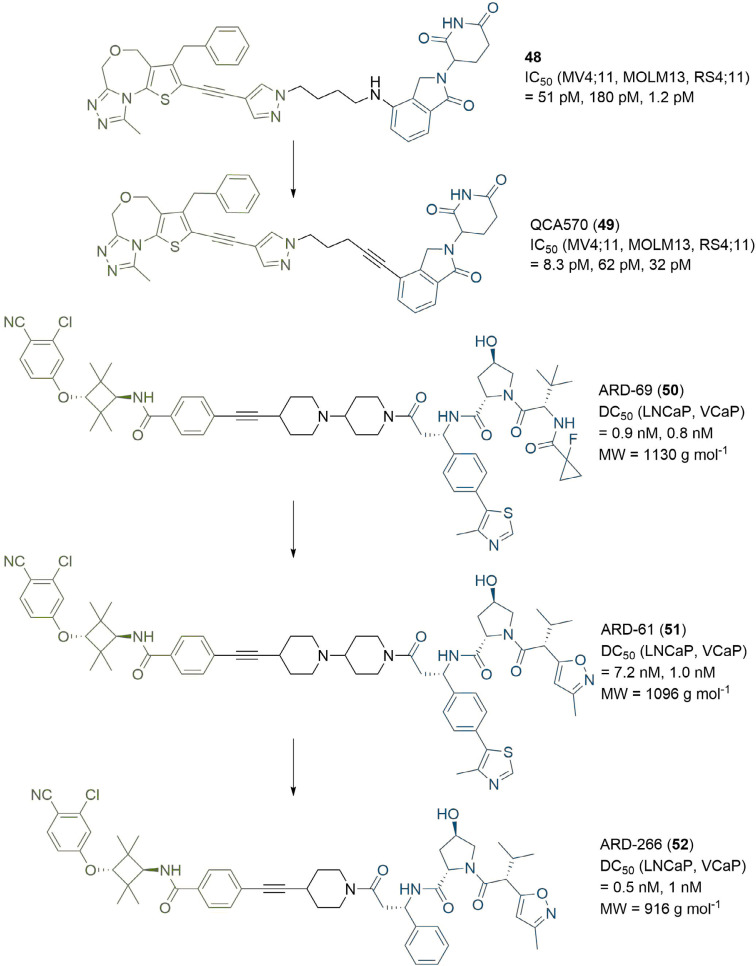
PROTACs with rigid linkers. Replacement of the amine connecting group to thalidomide in 48 with a rigid alkyne led to 49, which exhibited enhanced cell growth inhibition in 2/3 tested cell lines. Modifications to the anchor of 50 afforded 51, and further changes to the linker and anchor provided 52, which retained high degradation potency with a > 200 reduction in MW *vs.* 50

In recent studies, researchers have explored alternative linker strategies for the development of active PROTAC degraders. Linear alkyl and ether linkers are increasingly being replaced by motifs able to impart some rigidity, such as heterocyclic scaffolds (e.g., piperazine/piperidines) and alkynes, in addition to the incorporation of functional groups which are able to modulate the PROTAC physico-chemical properties. An example of the impact of PROTAC linker optimisation was described by the Wang group in 2018. In a series of BET degraders, the linker was initially optimised to a suitable length using alkyl chains, resulting in lead PROTAC 48 (Figure 9), which displayed IC_50_ values in the picomolar range in three leukaemia cell lines (MV4;11, MOLM13, and RS4;11) [77]. Replacement of the amine linkage to lenalidomide (11) with a rigid ethynyl group led to highly potent PROTAC QCA570 (49), displaying 3 and 6-fold increased cell activity compared to 48 in MOLM13 and MV4;11 cells respectively, but with a 27-fold potency decrease in the RS4;11 cell line (although still 32 pM inhibition). The authors did not comment on the underlying molecular basis for these observations, but this illustrative example is another testament to the importance of linker variation in PROTAC development. Employing a similar strategy, the same group developed the highly potent AR degrader ARD-69 (50), for potential treatment of metastatic castration-resistant prostate cancer [78]. Introduction of an ionisable pyridine/di-piperidine motif adjacent to the alkyne significantly improved aqueous solubility compared to parent PROTACs bearing all-hydrocarbon linkers. PROTAC 50, which contains a highly rigid linker, induced potent AR depletion (DC_50_ < 1 nM) in LNCaP and VCaP prostate cancer cell lines, along with downregulation of AR mediated transcription in the same cell lines. This highlights the benefit of employing rigid, polar linkers in contrast to traditional PEG/alkyl motifs as a strategy to improve pharmacokinetic properties, assuming that the rigid conformation is able to form a productive TC. A follow-up optimisation study of related PROTAC ARD-61 (51) led to ARD-266 (52) [79], which employs a VHL ligand with weaker (μM) binding affinity (Figure 9). Remarkably 52 retained subnanomolar AR degradation potency, hence highlighting that i) anchors targeting VHL with moderate affinity (i.e. micromolar) may be sufficient to achieve high cellular potency (i.e. nanomolar); and ii) potential strategies to mitigate off-target activity resulting from inhibition of hypoxia-inducible factors (HIF) signalling. Also of importance, the changes in the warhead and linker between 50 and 52 are accompanied by a significant (> 200) reduction in molecular weight (MW), reducing the gap between these PROTACs and traditional drug-like chemical space.

**Figure 10.**
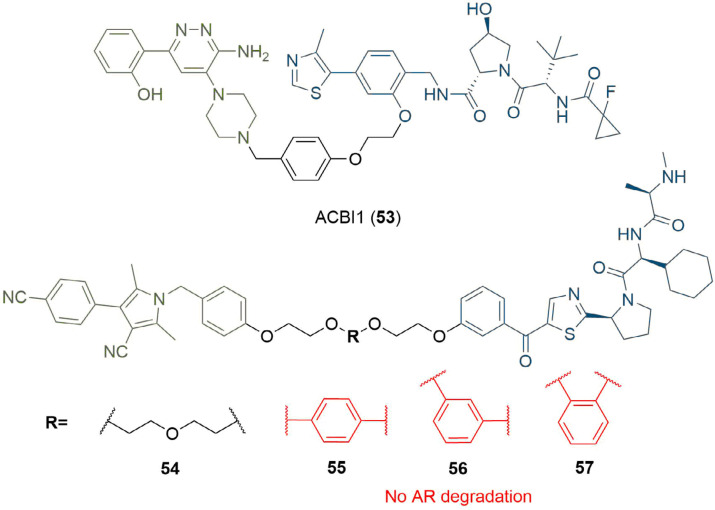
PROTACs with aromatic linkers. The benzyl linker in 53 provided conformational restriction and a pi-stacking interaction with Y98 in VHL. PROTACs 55-57 incorporating a disubstituted phenyl did not display AR degradation, in contrast to parent PROTAC 54

In the design of PROTAC degraders of the BRG1-associated factor (BAF) ATPase subunits SMARCA2 and SMARCA4, Farnaby et al. [80], linked a piperazine based SMARCA binding ligand to a VHL anchor using a benzyl linking fragment (Figure 10). In the resulting PROTAC ACBI1 (53), the para-disubstituted aryl unit was designed to provide conformational restriction, mimic the PEG linker conformation, and reduce the polarity of the PROTAC, in addition to targeting a specific pi-stacking interaction to a tyrosine residue in the VHL protein (Y98). However, in other cases, increasing linker rigidity has led to impaired degradation potency. Shibata et al. [81], substituted a PEG unit in their AR-targeting SNIPERs with a series of disubstituted phenyl rings to assess the effect of linker flexibility. In contrast to parent PROTAC 54, which exhibited AR degradation at 3 μM in 22Rv1 cells, none of the PROTACs 55-57 showed any activity against AR. This suggested that linear linked PROTAC 54 is able to adopt a productive conformation to enable TC formation and degradation, whereas PROTACs 55-57, containing aromatic groups are unable to induce degradation of AR.

### Clickable linkers

**Figure 11.**
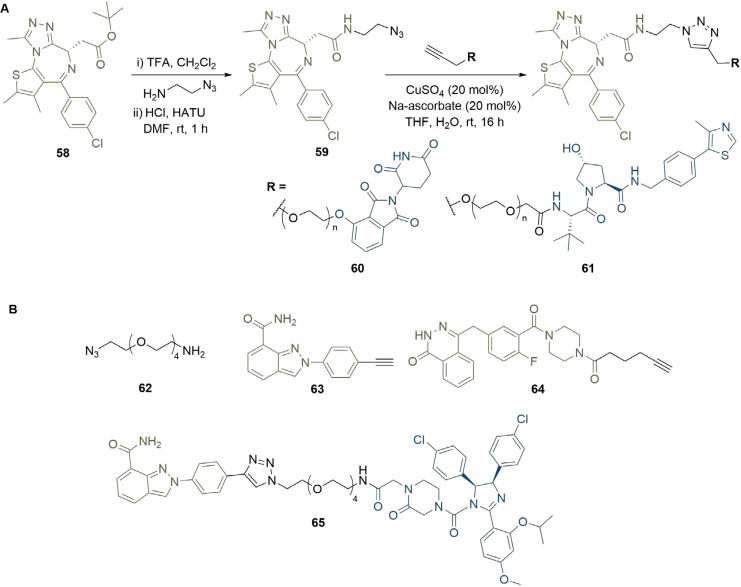
Use of triazoles in library synthesis. A. Azide intermediate 59 was reacted with alkynes bearing warheads for CRBN or VHL to afford two series of triazole-containing PROTACs 60 and 61 with variable linker lengths; B. amino azide intermediate 62 was conjugated to various anchors and reacted with alkyne derivatives of niraparib (63) or olaparib (64) to screen different warhead/anchor combinations. This identified potent MDM2-recruiting PROTAC 65

The triazole moiety appears commonly in reported PROTAC linker structures [82], which is due in part to the ease with which it can be installed using click chemistry, along with its chemical robustness to metabolism [83]. The copper-catalysed Huisgen 1,3-dipolar cycloaddition reaction between an alkyne and an azide has been extensively used to construct triazole motifs and generally proceeds in nearly quantitative yield, along with exceptional selectivity for the 1,4-disubstituted (anti) product [84]. This reaction has been used to expedite PROTAC synthesis in a highly convergent manner by using an alkyne moiety conjugated to one ligand and an azide conjugated to the other. This approach has proven valuable for optimising diverse libraries of PROTACs with variation in linker length, composition, site of conjugation, or conjugation vector [49, 85, 86]. Wurz et al. [87], probed the effects of linker length and ligase ligand on a series of BRD4 targeting PROTACs (Figure 11). Deprotection of the *tert*-butyl ester in JQ1 (58) followed by amide bond formation with 2-azidoethanamine provided warhead intermediate (59) with an azide handle. Conjugation of terminal alkyne linkers containing 0 to 4 PEG units (0 to 12 atoms) to VHL or CRBN targeting anchors, and final coupling of the two PROTAC halves by a Cu(I) catalysed click reaction, afforded a library of ten PROTACs in yields of up to 90% in the click step. Intriguingly, CRBN PROTACs (60) containing intermediate length linkers (1-2 PEG units) showed reduced BRD4 degradation potency (DC_50_ >5 μM) in H661 cancer cells compared to those with shorter and longer linkers (0, 4-5 PEG units, < 0.5 μM). This unexpected pattern was not replicated in the VHL series (61), in which potency decreased as linker length increased, and further highlights the crucial requirement to optimise linker length for each ligand pair when designing PROTACs. Triazole click chemistry has also been used for the combinatorial PROTAC synthesis and rapid identification of anchor-linker-warhead combinations displaying optimal degradation efficiency. Zhao et al. [88], generated a series of potential PARP1 degraders by conjugating the same acid and azide functionalised linker intermediate (62) to either a niraparib (63) or olaparib (64) derived warhead and a ligand for VHL, CRBN or MDM2. Their linker contained an amine linked to the anchor through amide bond formation or S_N_Ar, and an azide that could be coupled to the alkyne moieties in 63 or 64 through click chemistry (Figure 11). Lead PROTAC (65), combining the MDM2 ligand nutlin-3 (14) with 63, induced potent PARP1 cleavage and apoptosis in the MDA-MB-231 breast cancer cell line, which is likely due to PARP1 degradation.

**Figure 12.**
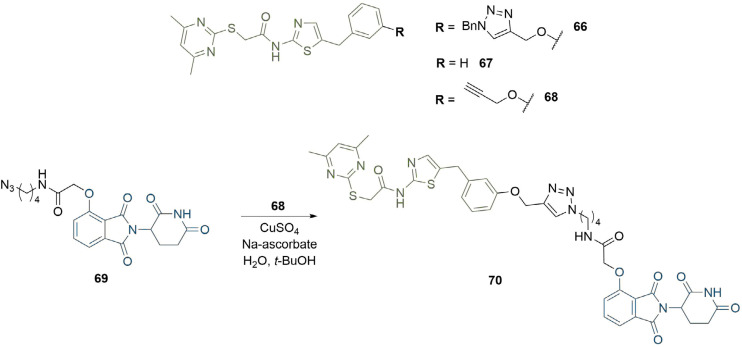
Use of triazoles to exploit intermolecular interactions. The nitrogen atoms in the triazole formed hydrogen bonds with R97 in Sirt2 in the crystal structure of 65. Following the click reaction with azide 69, triazole-containing PROTAC 70 retained these interactions in docking analysis of the TC

Triazoles can have utility beyond simply facilitating the synthesis of PROTACs; they can also be harnessed to modulate physical properties, or to exploit new intermolecular interactions to stabilise the TC. In the development of a sirtuin rearranging ligand (SirReal) based probe compound, Schiedel et al. [89], identified triazole functionalised SirReal analogue 66, which exhibited improved aqueous solubility compared to its parent compound (67). The co-crystal structure of 66 in complex with Sirt2 (PDB 5DY5) revealed that the triazole ring extends into the binding channel for acetyl lysine and picks up a hydrogen bond to arginine residue R97 of Sirt2. Further, N1 of the triazole unit is solvent exposed and thus could be used as a linker attachment point in PROTAC construction. Therefore, the motif was retained for the development of Sirt2 degraders. The alkyne functionalised SirReal ligand (68) was conjugated to the azide functionalised thalidomide derivative (69) through the copper catalysed Huisgen cycloaddition to produce lead PROTAC 70. Docking analysis of 70 in the TC with Sirt2 and CRBN suggested that this hydrogen bonding interaction was conserved, and that the overall binding to Sirt2 was very similar to that of the free ligand (Figure 12).

**Figure 13.**
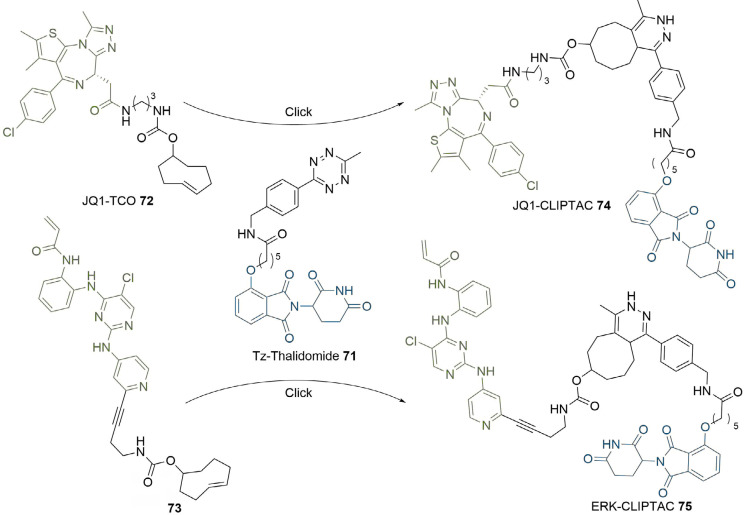
*In-situ* formation of CLIPTACs 74 and 75 by the click reaction between tetrazine 71 and TCO 72 or 73

The use of click chemistry was taken a step further by Lebraud et al. [90], who developed alkyne and tetrazine precursor molecules for the in-cell self-assembly of PROTACs, termed CLIPTACs. This strategy aimed to alleviate cell permeability and solubility issues resulting from the high MW and large topological polar surface area (TPSA) of most degraders. The strategy is based on the hypothesis that lower MW precursors bearing “clickable” groups for *in cellulo* assembly would likely display better cell membrane permeability than the corresponding PROTAC. Their approach relied on the bioorthogonal inverse electron demand Diels-Alder reaction between a tetrazine and *trans*-cyclooctene (TCO) fragment as their *in cellulo* PROTAC-forming step [91]. To do this, they utilised a tetrazine functionalised thalidomide derivative (Tz-thalidomide, 71) and TCO derivatives of BET ligand JQ1 (72) and a covalent extracellular signal-regulated kinase (ERK)1/2 inhibitor (73, Figure 13). No assessment of the effect of linker length on degradation was reported in this work. Analysis of the x-ray crystal structure of thalidomide in complex with CRBN (PDB 4CI1) when designing 71, allowed determination of the minimum linker length that would place the tetrazine moiety into the solvent [92]. While the resultant linker in JQ1-CLIPTAC (74) is longer than in highly potent ARV-825 (2), computational analysis suggested that its nonlinear shape would place the anchor and warhead of 74 at a similar distance. A comparison of clickable precursors 71, 72, and 73 to published BRD4 degraders 1-3 confirmed that they had significantly reduced MW and TPSA, although lipophilicity of the TCO derivatives is significantly higher (Table 2).

**Table 2.** Comparison of the physical properties of CLIPTAC precursor molecules (71–73) to published BET degraders (1–3)

**Compound**	**MW**	**cLog*P***	**TPSA**
71	572	1.2	173
72	609	5.9	111
73	586	6.5	130
MZ1 (1)	1003	4.9	211
ARV-825 (2)	924	4.8	205
dBET1 (3)	785	2.5	194

When administering 71 and 72/73 sequentially, *in situ* formation of JQ1-CLIPTAC 74 exhibited complete depletion of BRD4 in HeLa cells after 24 h, while ERK-CLIPTAC 75 showed similar performance and completely depleted ERK1/2 in A375 cells after 16 h (10 μM of both clickable components used in each case). Crucially, administering cells with identical concentrations of pre-formed CLIPTACs did not result in any degradation. This is in line with the generally low cell permeability of other PROTACs, and confirmed that the *in cellulo* clicking of the two precursors was leading the degradation. However, one significant drawback of this method is that cells must be treated sequentially with each precursor to prevent rapid clicking outside of cells.

### Photoswitchable linkers

**Figure 14.**
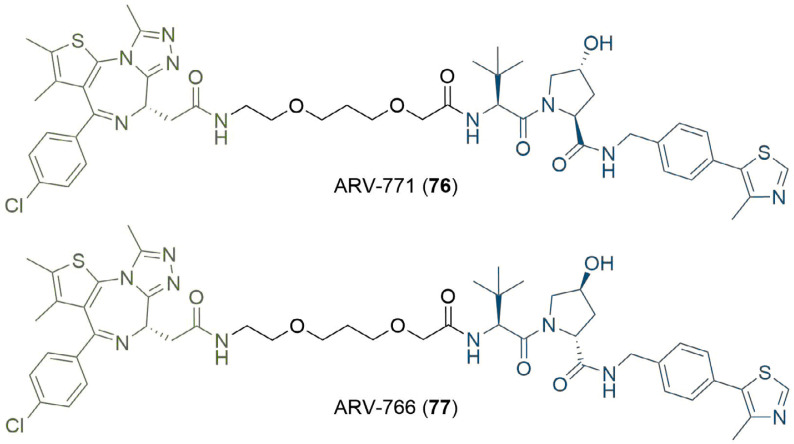
Structures of potent BET degrader ARV-771 (76) and its inactive epimer ARV-766 (77)

Whilst the potential therapeutic applications of PROTACs have been well documented, there is some evidence that the systemic application of PROTACs can have undesirable effects. For example, the potent BET degrader ARV-771 (76) has been shown to achieve tumour regression in a castration-resistant prostate cancer mouse xenograft model and validates the development of BET degraders as a potential therapeutic strategy [93]. However, Raina et al. [93], observed various toxicities when dosing with 76, which did not occur when the inactive epimer ARV-766 (77) was administered (Figure 14). Skin discolouration was observed at the injection site, but this was found to be reversible after a 2–3 day dosing holiday. More concerning effects were observed with intermittent dosing of the mice (daily dosing was not tolerated), such as a reduction in their physical activity levels, as well as spinal deformities. The mechanism of these associated toxicities is unclear but may not be due to a PROTAC-specific liability; the suppression of BRD4 has been shown in an RNAi mouse model to cause reversible epidermal hyperplasia and alopecia amongst other effects [94], but these results do still highlight a potential benefit to PROTACs that can be controlled in a spatiotemporal manner. Several groups have investigated this approach recently using light stimuli to elicit this control, due to the high precision with which this can be applied [95].

**Figure 15.**
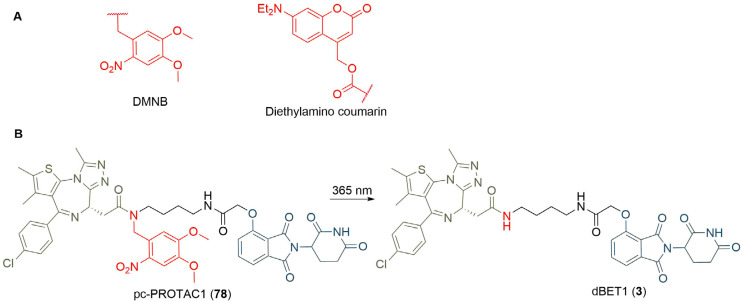
A. Structures of photocleavable groups DMNB and diethylamino coumarin; B. irradiation of DMNB-protected PROTAC 78 at 365 nm releases potent BET degrader dBET1 (3)

One approach to photoswitchable PROTACs is to incorporate a photolabile “cage” into the PROTAC structure. In the presence of light, this group is cleaved, and the active PROTAC is released. An example of this is the 4,5-dimethoxy-2-nitrobenzyl (DMNB) group, and “opto-PROTACs” have been reported with DMNB installed on the glutarimide NH of pomalidomide (10) [96], and the hydroxyl group of the VHL ligand (8) [97]. In both cases, the presence of the DNMB group inactivates the PROTAC by preventing binding to its respective E3 ligase. A photolabile diethylamino coumarin group has also been employed for the same purpose (Figure 15) [98]. Xue et al. [99], incorporated the DMNB group onto the amide nitrogen of dBET1 where the linker connects to JQ1 to obtain pc-PROTAC1 (78). This alteration reduced the binding affinity of 78 for BRD4 by more than 100-fold, correlating with a lack of appreciable BRD4 degradation in Ramos cells when incubated in the dark. Irradiation with UV light at 365 nm cleaved the DMNB group and released dBET1 (3), leading to almost complete BRD4 degradation (D_max_ = 93%) at 1 μM.

**Figure 16.**
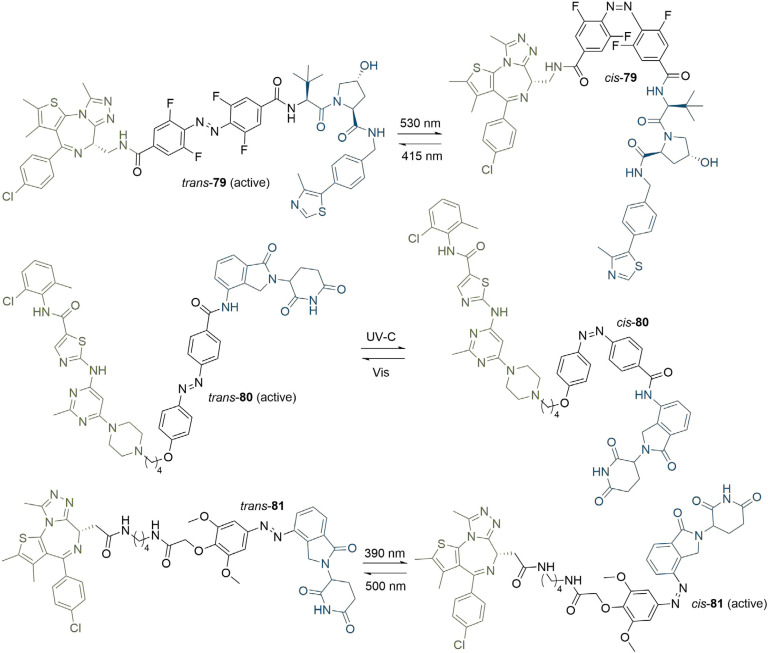
PROTACs with photoswitchable linkers. PROTACs 79 and 80 are active degraders in their *trans* configuration, whereas 81 is active in its *cis* configuration. The wavelengths required for conversion between the *trans* and *cis* forms are given above and below the arrows

Another method to achieve light controlled PROTACs that has been developed concurrently by the Trauner, You/Jiang, and Crews/Carreira groups is the incorporation of a photoswitchable element into the linker (Figure 16). Each approach employed azobenzenes as the photoswitch, which can reversibly undergo *cis*-*trans* isomerisation upon irradiation at different wavelengths. Pfaff et al. [100], identified that the difference in linker length between the *trans* and *cis* azobenzene isomers was approximately 3-4 Å, which has a great similarity to the critical difference in linker length between active and inactive degraders for several published PROTACs (around 3 Å). They used an ortho-F_4_-azobenzene to generate a bistable “photoPROTAC” (79), which could be switched between photostationary states (PSS) by irradiation at 415 nm or 530 nm. Irradiation at 415 nm gave rise to a PSS with 95% *trans*-79, whilst irradiation at 530 nm led to a PSS with 68% *cis*-79. The authors did not observe any thermal back-isomerisation of *cis*-79 under biological conditions over several days. After sample irradiation at 415 nm, *trans*-79 induced significant BRD2 degradation in Ramos cells at low micromolar concentrations, while inducing comparatively low BRD2 degradation with irradiation at 530 nm in the same concentration range. In contrast to ARV-771 (76), *trans*-79 could not induce BRD4 degradation despite employing the same anchor/warhead pair and containing a similar length linker. The underlying reasons are unclear, however this may be partly explained by potential cooperativity in the BRD2 TC, along with the genomic and proteomic variations between Ramos cells and the separate cell lines (22Rv1, VCaP, and LnCaP95) in which ARV-771 was previously evaluated. Earlier this year, Jin et al. [101], developed photoswitchable “azo-PROTAC” degraders for the BCR-ABL fusion and ABL proteins. These were also active in their *trans* form and inactive when *cis*. The rationale behind this design was based on the analysis of the X-ray crystal structure of CRBN in complex with lenalidomide (11, PDB 4TZ4). The relatively small and narrow nature of the lenalidomide binding pocket suggested that linking the diazobenzene moiety directly to lenalidomide would lead to a steric clash between the protein and the rest of the PROTAC in its *cis* configuration, and so be inactive. This approach was validated, as they observed that only *trans* azo-PROTAC 80 was an effective degrader. In contrast to the other photoswitchable PROTACs, those developed by Reynders et al. [102], (termed “PHOTACs”) were active BET degraders in their *cis* configuration. When irradiated with 390 nm light, representative PHOTAC 81 rapidly isomerised to a PSS with > 90% of the *cis* form and displayed cytotoxicity in RS4;11 lymphoblast cells. In cell viability assays, they observed a 7 fold difference in EC_50_ between a sample of 81 irradiated at 390 nm (89 nM) and another kept in the dark (631 nM), where the *trans* form was predominant due to thermal back-isomerization. The *cis* state of 81 displayed limited stability, and isomerised to the *trans* configuration with a half-life of 8.8 h, hence requiring continued light pulses to maintain prolonged degradation.

A summary of the linker motifs described in this section is provided in Table 3.

**Table 3.** Key considerations for linker motifs described

**Structure**	**Linker type**	**Key points**
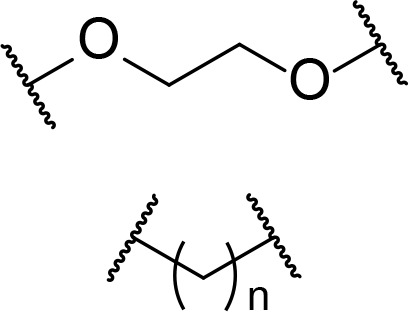	Alkyl/PEG	- High synthetic accessibility and commercial availability - Enable fine-tuning of linker length - Flexible
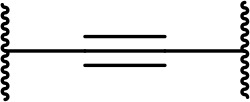	Rigidifying groups	- Potential potency improvements - More favourable physical properties - Conformational restriction
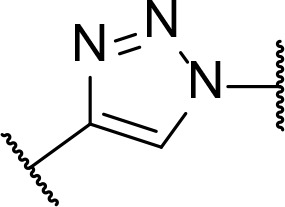	Clickable groups	- Facilitates library synthesis - High-yielding synthesis - Potential H-bond interactions in the TC
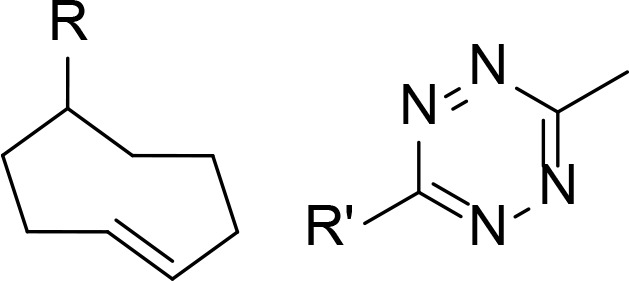	CLIPTACs	- Assembled from lower MW precursors - More favourable physical properties - Compounds must be administered separately to avoid clicking
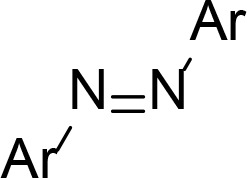	Photoswitches	- High spatiotemporal control - May alleviate toxicity - Continuous irradiation may be required if photostates are not bistable

## Linker design strategies

A significant proportion of reported degraders were developed through mostly empirical optimisation of linker composition, often driven by the commercial availability of the linker precursor. Empirical PROTAC linker optimisation usually requires the synthesis of large libraries of compounds containing linkers of various compositions, lengths, and with different connecting functional groups [103]. As an illustrative example, Zhang et al. [104], synthesised over 50 PROTACs in their search of a potent, selective, and bioavailable degrader of the anti-apoptotic protein BCL-X_L_. This included the parallel development of two PROTAC series, one targeting VHL and the other CRBN, but the warhead and anchor ligands were fixed in both. Concurrent linker optimisation in each series was required as the optimal linker for one warhead/anchor pair usually does not translate across different E3 ligase anchors. Similarly, Su et al. [105], synthesised over 40 PROTACs in their pursuit of potent and selective CDK6 degraders as they screened different combinations of warhead, linker, and anchors for four E3 ligases (VHL, CRBN, cIAP, and MDM2). Such combinatorial approaches are generally resource intensive, and sometimes include little element of rational design. This has been increasingly recognised, and recent years have seen a significant shift towards methods to design PROTAC linkers more rationally and focus synthetic efforts. In particular, an important emphasis has recently been on modifying the linker structure in line with physical property optimisation, exploiting information gained from available crystal structures or computationally generated homology models to identify suitable points for linker attachment or opportunities to gain new interactions in the TC.

### Consideration of physical properties

The chemical composition of the linker has a significant impact on the physico-chemical properties of the PROTAC molecule, which in turn has implications for its pharmacokinetic profile [106]. Due to their high MW, PROTACs invariably lie in chemical space beyond the guidelines of Lipinski’s rule-of-5 for achieving good oral bioavailability [107], although there are examples of orally available degraders [108] and many examples of beyond rule-of-5 oral drugs [109, 110]. PROTACs also tend to possess high TPSAs and large numbers of rotatable bonds (nRotB) which place them outside of similar guidelines from Veber [111]. Metrics such as the multiparametric scoring function AB-MPS have been developed to provide guidelines for development of compounds in beyond rule-of-5 space (a lower AB-MPS score indicates a higher likelihood of absorption) [112], and recent analyses of these compounds have provided new descriptors to predict absorption [113, 114]. Edmondson et al. [115], used the AB-MPS score alongside various *in silico* metrics associated with permeability/absorption [hydrogen bond donors and acceptors (HBDs and HBAs), polar surface area (PSA), nRotB, Nrule-of-5, nAr, and cLog*P*/*D*] to analyse a representative set of 38 PROTACs across the four most commonly recruited classes of E3 ligase (VHL, CRBN, cIAP, and MDM2). The properties of the anchor had a noticeable effect on the overall PROTAC properties: MDM2 and cIAP-recruiting PROTACs generally possessed high MW, lipophilicities and AB-MPS scores, which indicates that oral absorption may be challenging for these classes. VHL PROTACs scored better in these metrics, and CRBN PROTACs better still, due to the more favourable properties of the smaller thalidomide analogues when compared to the VHL ligand. Despite the known issues associated with thalidomide’s rapid epimerisation and instability in cells [116], CRBN based PROTACs were found to be closer to “drug-like” chemical space on average, although all PROTAC classes suffered from high numbers of HBD and nRotB mainly driven by the prevalence of long, linear PEG and alkyl linkers in the PROTACs discussed. The authors also highlighted potential concerns around potential oxidative metabolism of linkers with linear aliphatic or ether chains, especially given the high lipophilicities of most compounds in their dataset. The toxicity and metabolic stability of PEG and their conjugates have been extensively discussed [117–119]. Since the warhead and anchor are often fixed in PROTAC development, optimisation of the linker moiety provides an opportunity for increased degradation efficiency (e.g., via increased TC cooperativity), in addition to providing a handle for the modulation of physico-chemical parameters and ultimately controlling the drug metabolism and pharmacokinetic (DMPK) profiles of PROTAC degraders. In their assessment of the DMPK optimisation of PROTACs, Cantrill et al. [120], stated the critical importance of solubility as a parameter to optimise to generate oral degraders. They argued that permeability, another key factor in determining overall DMPK profile, is difficult to improve due to the nature of the PROTAC mode of action, which necessitates high MW compounds. The linker provides the most likely route to optimise solubility, such as by replacement of more traditional alkyl and PEG moieties with saturated nitrogen heterocycles or other polar rigidifying groups, which could also benefit permeability. Finally, Maple et al. [62], defined their own metric to evaluate PROTAC performance by assessing degrader score (Deg_S) efficacy. This score is calculated by the summation of various parameters for a given PROTAC, normalised against the total number of parameters used.

$$\text{Deg}\_\text{S}=\frac{\left(x_{d}+x_{m}+x_{o}+x_{c}+x_{t}\right)}{n_{U}}$$

Where *x_d_* is the DC_50_ (nM), *x_m_* is the D_max_ (%), *x_o_* is the percent of observed degradation, *x_c_* is the degrader concentration (μM), *x_t_* is the incubation time (h), and *n_U_* is the number of parameters. A score from 1 to 7 is applied to each metric based on its standardised value (e.g., 0 < *x_d_* ≤ 30 scores 7). When analysing degraders in the CRBN and VHL-recruiting classes, they noted that increasing Deg_S was correlated with increasing cLog*P* and decreasing TPSA and HBD count. While there is generally limited scope for altering the anchors and warheads, altogether these studies suggest that PROTAC property modulation through synthetic alterations of the chemical composition of the linker could provide exciting opportunities for bringing PROTACs into a more acceptable chemical space for oral absorption and improve general bioavailability.

**Figure 17.**
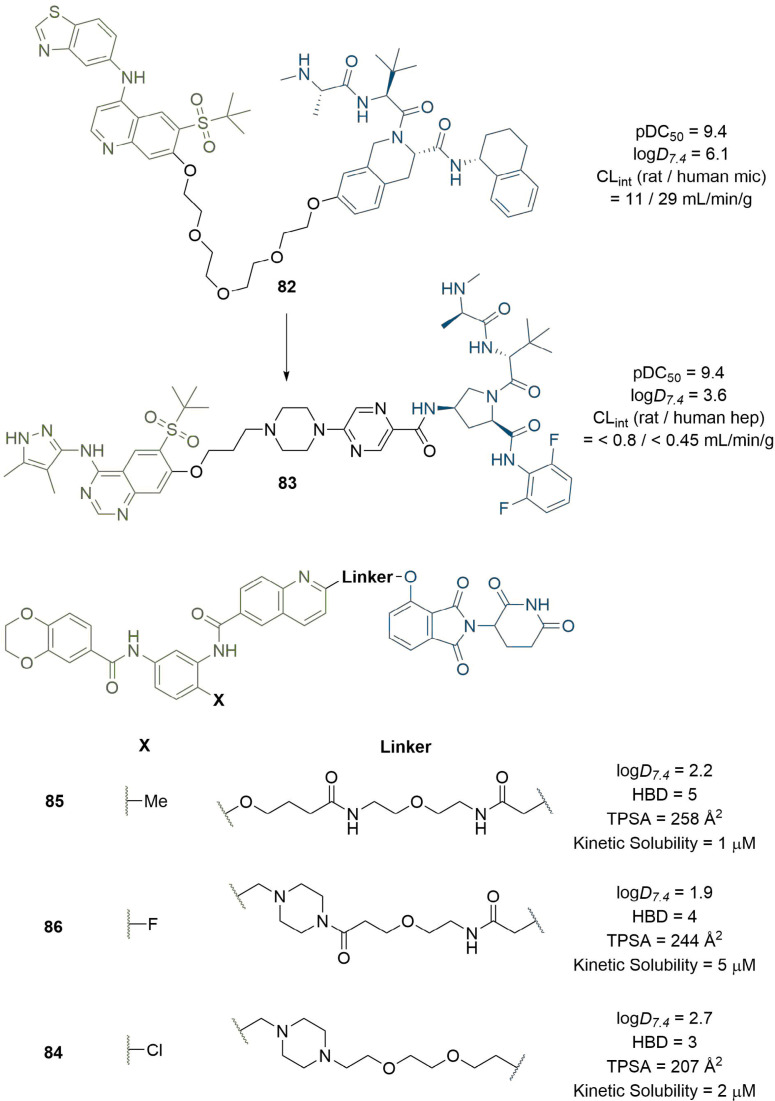
PROTACs with linkers optimised to improve physical properties. Replacement of the PEG linker in 82 with piperazine and pyrimidine moieties (alongside other changes) greatly reduced lipophilicity and metabolic clearance of 83. Chessum et al. [126], developed pirin-targeting probe 84 in only three focused design iterations (through 85 and 86) by seeking to optimise physical properties instead of potency

Recently, an increasing number of publications have emerged where the physical properties of PROTACs are accounted for in their design, or in the rationalisation of their efficacy [121–124]. Mares et al. [125], developed a potent cIAP-recruiting PROTAC (82) for the degradation of RIPK2 containing a PEG linker (pDC_50_ = 9.4 in THP-1 monocytes, Figure 17). However, compound turnover in rat and human microsomes was high (11 and 29 mL/min/g liver respectively) and solubility was poor, which limited its utility as an *in vivo* tool molecule. The ChromLog*D*_7.4_ of 82 was reduced from 6.1 to 3.6 through modification of all three components of the PROTAC: pyrazole replacement of a benzothiazole ring in the warhead, replacement of the IAP-recruiting anchor, and incorporation of polar piperazine and pyrimidine moieties into the linker. The resulting PROTAC (83) maintained the high potency of 82 (pDC_50_ = 9.4) but exhibited far lower metabolic clearance (< 0.8 and < 0.45 mL/min/g liver in rat and human hepatocytes respectively) and improved aqueous solubility (346 μM). Chessum et al. [126], developed pirin-targeting protein degradation probe (PDP) CCT367766 (84), having previously reported a high affinity benzodioxine based ligand for pirin. Their first generation PDPs did not elicit degradation so required optimisation, and they acknowledged that the requisite synthesis of analogue libraries to probe different E3 ligases, linker lengths, and compositions probe SAR would be lengthy and challenging. They hypothesised that the PDP’s physico-chemical properties were mainly responsible for the poor performance of their first generation probe 85, and envisaged that solely optimising linker composition may be sufficient to maximise cell membrane flux and enhance degradation, whilst keeping the length, warhead, and anchor constant (Figure 17). They aimed to reduce the calculated TPSA (258 Å^2^) and HBD (5) count of 85 whilst maintaining its acceptable Log*D*_7.4_ (2.2). Substitution of the ether linker for a methylene piperazine, and bioisosteric replacement of Me for F in the warhead to reduce HBD count by masking the interaction of the nearby amide afforded PROTAC 86. This compound exhibited complete pirin degradation at 3 μM in SK-OV-3 ovarian cancer cells after 24 h, but this required concentrations close to its kinetic solubility (5 μM). Bioactivity was also limited by the poor cellular stability of 86 (t_1/2_ ~4 h) consistent with the known decomposition of the CRBN-targeting moiety [127]. Increasing permeability to mitigate this instability by bioisosteric replacement of F for Cl, followed by lipophilicity adjustment by substituting the tertiary amide to the piperazine to the corresponding ionisable amine afforded PROTAC 84. This displayed a significantly reduced TPSA (207 Å^2^ *vs.* 258 Å^2^ in 85) and elicited near complete pirin degradation after 2 h at 50 nM concentrations. This is a remarkable example illustrating how focusing on the optimisation of physical properties rather than simply affinity can drastically enhance degradation efficiency, in this case in only three focused iterations.

### Exploiting x-ray crystal structures of protein/ligand binary complexes

**Figure 18.**
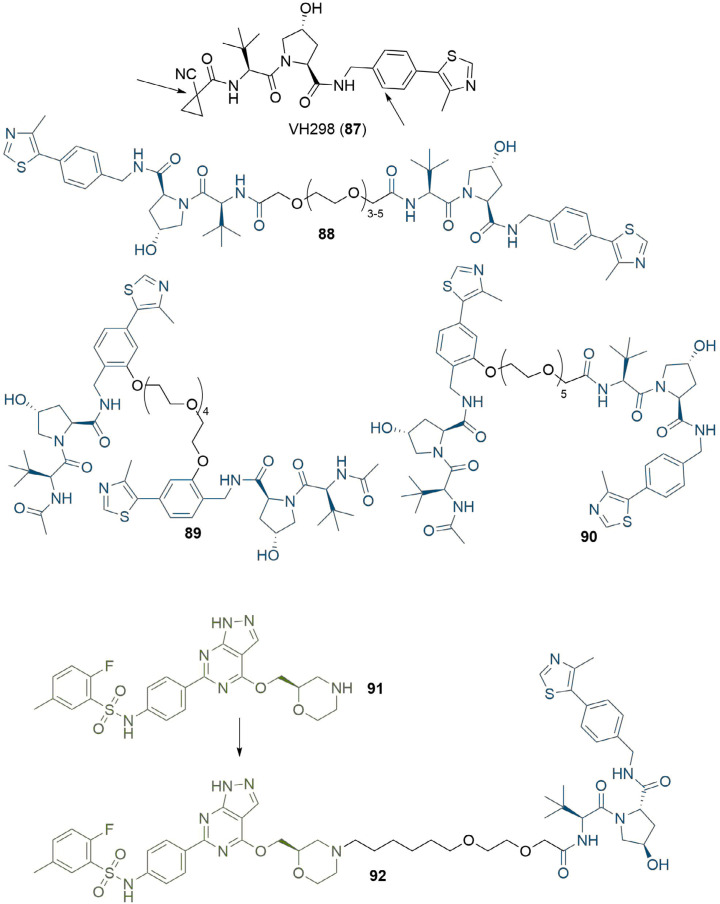
Use of co-crystal structures to identify linker conjugation sites. Arrows indicate sites on VH298 (87) that are solvent exposed. This was used to inform the design of three combinations of analogous VH032 (8) in homo-PROTACs 88-90. The morpholine nitrogen in SGK3 inhibitor 91 was shown to be solvent-exposed, and was used for linker conjugation in PROTAC 92

It is well documented that the identification of appropriate vectors from the warhead and anchor is critical to devise a suitable strategy for their conjugation to the linker and access potent degraders [128]. The availability of high-resolution co-crystal structures for warheads bound to the POIs is an invaluable tool and often a prerequisite for PROTAC design and assembly, in particular to identify solvent-exposed exit vectors on the warhead where a linker can be conjugated with minimal effect on POI binding [129–132]. As an illustrative example, Maniaci et al. [133], utilised the co-crystal structure (PDB 5LLI) of VHL in complex with VH298 (87) for the development of “Homo-PROTACs” for the self-induced degradation of VHL. Visual analysis highlighted two solvent-exposed positions where the analogous VHL ligand 8 could be derivatised without affecting its binding mode (Figure 18). They synthesised PROTACs 88–90 with the three combinations of different attachments points, and found that the most active PROTACs were symmetrically linked from the terminal left hand side acetyl group of 8 (88). Derivatisation at other positions led to ineffective degraders, which underlines the importance of appropriate linkage position. In the absence of a co-crystal structure, the choice of conjugation site must instead be made through SAR studies. In the development of serum- and glucocorticoid-regulated kinase family member 3 (SGK3) PROTACs, Tovell et al. [134], exploited SAR data previously disclosed by Sanofi on small molecule SGK inhibitors. They identified that aliphatic and cyclic substituents at the 4-position of the pyrazolopyrimidine core were well tolerated, and hence hypothesised that this region could be solvent exposed. The morpholine functionalised inhibitor 91 was elected for PROTAC assembly due to its nitrogen handle allowing straightforward *N*-alkylation, and eventually identified highly selective and potent SGK3 degrader 92 after linker optimisation (Figure 18).

**Figure 19.**
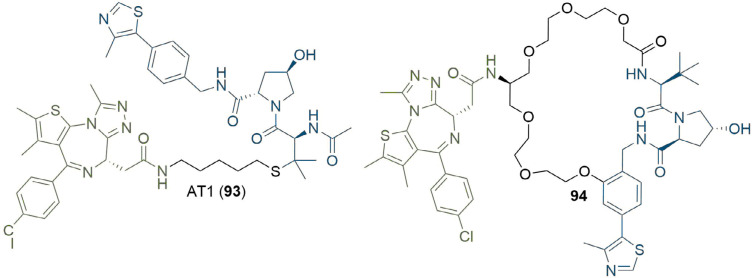
Rational design of PROTACs from TC crystal structures. The crystal structure of MZ1 (1) suggested that the *tert*-butyl moiety was a better site for linker conjugation, which was used to product AT1 (93). Macrocyclisation of 1 to retain its binding conformation in the TC crystal structure afforded 94

The publication of the crystal structure of MZ1 (1) in complex with VHL-ElonginC-ElonginB (VCB) and BRD4^BD2^ (PDB 5T35) has provided additional means for researchers to design linker structures rationally [54]. In the complex, MZ1 is “sandwiched” between the two proteins, with folding of the linker facilitating this. In addition to new PPIs, the PEG linker engages in a hydrogen bond with a histidine residue specific to BD2 (H437), along with extensive van der Waals interactions with the BC loop of BRD4^BD2^. These new contacts suggested that isoform-specific interactions could increase the cooperativity (α) of the TC and provide a blueprint towards generalisable approaches aimed at improving the potency and selectivity of PROTACs by exploiting TC specific interactions [135–137]. Isothermal titration calorimetry (ITC) was used to probe the thermodynamics of TC formation in response to 1 and identified that BRD4^BD2^ and BRD3^BD2^ displayed the highest positive cooperativity of TC formation among all BET domains (α = 17.6, 10.7 respectively) and also formed the most stable TCs (ΔG = −22 kcal/mol). Furthermore, the crystal structure also indicated that the *tert*-butyl group of VHL anchor 8 may provide a better vector for linkage to MZ1, which they exploited to produce PROTAC AT1 (93) (Figure 19). Interestingly, 93 exhibited greater cooperativity in TC formation with BRD4^BD2^ (α = 7) and was more selective than MZ1; it potently degraded BRD4 in HeLa cells at sub micromolar concentrations with comparatively negligible degradation of BRD2/3. This is an important example of bioactivity enhancement through varying the exit vectors on the anchor and the warhead. Perhaps more notably, this highlights the importance of considering binding cooperativity during biophysical SAR studies, and the potential use of α as a quantitative indicator for the rational selection of best *in vitro* PROTAC lead(s) for cellular studies. In related work, the Ciulli group used macrocyclisation as a strategy to lock MZ1 in its bound conformation as a way to enhance the energetic bias for productive TC formation (Figure 19) [138]. Visual inspection combined with molecular modelling were used to choose a suitable conjugation vector and linker length between a phenolic group on a Hyp based VHL ligand and the first methylene of the PEG chain. This “macro-PROTAC” (94) exhibited positive cooperativity in formation of TC with BD2 of BRD2-4 (α = 9.5, 4.0, and 10.5 for BRD2, BRD3 and BRD4 respectively), but no cooperativity with BD1 (α < 1 in all cases), suggesting better differentiation between the two bromodomains than its parent MZ1 (1). We have previously reported structural evidence that > 10-fold selectivity for the second BET bromodomain can be achieved by exploiting subtle amino acid changes in the BC loop flanking the warhead binding site. In particular, an aspartate residue in the BC loop (Asp160 in BRD2^BD1^) is conserved among all first BET bromodomains and conservatively replaced by a histidine residue in the second BET bromodomains (His433 in BRD2^BD2^) [139]. Similar to MZ1, 94 showed selective BRD4 degradation (DC_50_ between 25 and 125 nM in 22RV1 human prostate carcinoma cells) but still degraded BRD2/3 at higher concentrations (DC_50_ > 125 nM).

**Figure 20.**
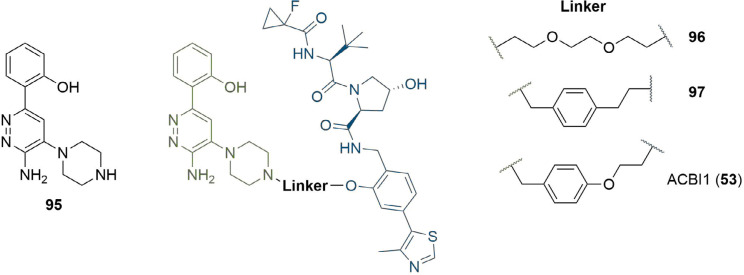
Use of co-crystal structure to guide changes to linker composition. The crystal structure of 95 in complex with SMARCA2^BD^ identified the piperazine ring as suitable for conjugation. The PEG linkage in 96 was replaced with a benzyl to improve hydrophobic interactions and exploit a potential pi-stack to Y98 in VHL (97). Extension by one atom to obtain the same length as 96 afforded 53

The availability of a co-crystal structure can be used for the rational design of new PROTACs by identifying changes that can be made to the ligands or linker to gain new intermolecular interactions [48]. This approach was used by Farnaby et al. [80], in the development of a potent degrader of SMARCA2, SMARCA4, and PBRM1 in only two design iterations (Figure 20). They solved the co-crystal structure of SMARCA ligand 95 in complex with the bromodomain of SMARCA2 (PDB 6HAZ), and identified that the solvent-accessible piperazine ring was likely a suitable linker conjugation point. Synthesis of an initial set of PROTACs with PEG linkers identified 96, which displayed positive cooperativity in the formation of a TC with VCB and SMARCA2 (α = 4.8), although it was only able to induce partial degradation of SMARCA2 and SMARCA4 in MV-4-11 cells (D_max_ = 65, 70% and DC_50_ = 300, 250 nM respectively). Permeability was shown to be low (1.1 × 10^−7^ cm s^−1^) and efflux ratio high (190:1), which suggested that degradation may be limited by cellular permeability. The high-resolution co-crystal structure (PDB 6HAY) of the TC formed by VCB and SMARCA2^BD^ with 96 was used to inform potential changes to the linker that could lead to a more effective and permeable degrader without lengthy empirical optimisation. The authors noted that the flexible PEG linker collapsed onto a hydrophobic region created by a tyrosine residue (Y98) in the VHL protein, and sought to optimise this interaction. They introduced a 1,4-disubstituted phenyl ring into the linker to form a pi-stacking interaction to Y98, reduce the polarity of the linker, and to increase conformational restraint whilst maintaining the same overall geometry. Co-crystallisation of this optimised PROTAC (97) with VCB and SMARCA2^BD^ (PDB 6HAX) showed a T-stacking interaction between the linker phenyl ring and Y98, confirming that the requisite linker conformation was maintained. Improvements in permeability (8.4 × 10^−7^ cm s^−1^) and efflux ratio (9:1) were also observed in this compound. Introduction of an oxygen atom into the linker to bring it to the same length as in 96 yielded ACBI1 (53, Figure 10). ACBI1 showed further improvements in permeability and efflux ratio (2.2 × 10^−6^ cm s^−1^, 1.7:1) and demonstrated a high degree of cooperativity in TC formation (α = 30). In contrast to parent PROTAC 96, PROTAC 53 showed complete SMARCA2 and SMARCA4 degradation in MV-4-11 cells (DC_50_ = 6 nM and 11 nM respectively). By using crystal structures of their PROTACs in TC formation, the authors were able to develop a potent SMARCA2/4 degrader in only three design iterations.

### Computational methods

**Figure 21.**
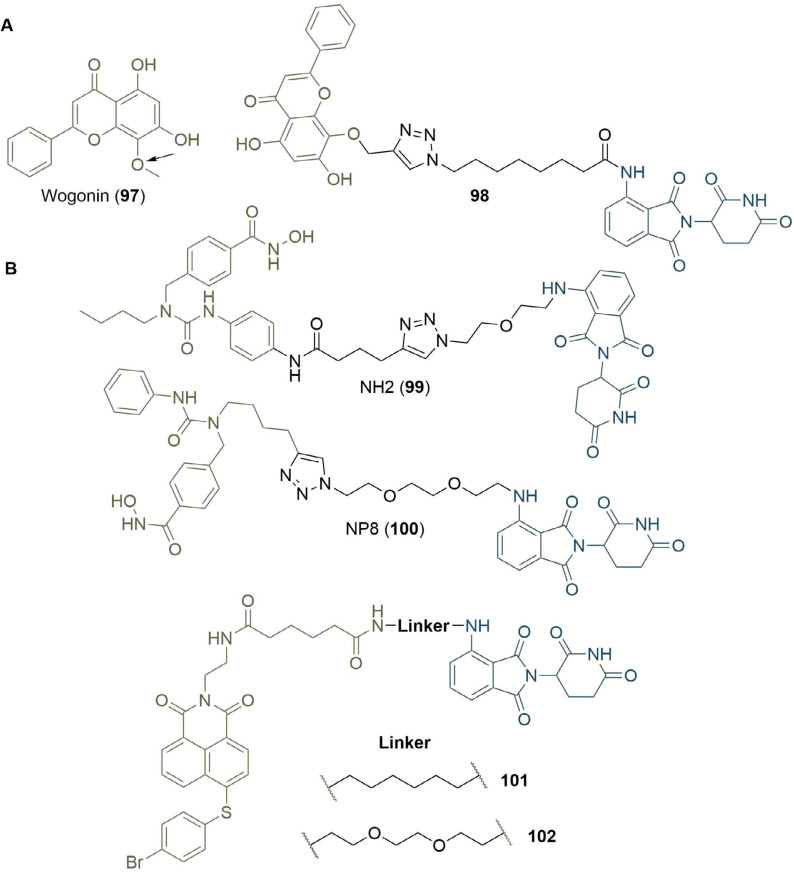
Use of computational docking to inform rational linker design. A. Docking pose of wogonin (97) was used to identify suitable conjugation vectors whilst retaining key binding interactions (site indicated by arrow), which was achieved in PROTAC 98; B. experimental SAR studies combined with computational docking was used to probe plausible TC ensembles formed by HDAC6 degraders 99 and 100, and suggested that each degrader employed a different set of amino acids to form distinct productive TCs. Similarly, modelling of the TC using a combination of docking and molecular dynamics was used to explain the orthogonal selectivities displayed by 101/102 towards MCL-1/BLC-2

Along with visual inspection of crystal structures, computational modelling of PROTACs in binary or ternary complexes has been increasingly used to rationally design PROTAC linkers. Where crystal structures of the warhead ligand in complex with the POI are unavailable, computational methods can be utilised to determine appropriate sites and vectors for linker conjugation [140, 141]. Bian et al. [142], used the docking pose of the natural product wogonin (97) in complex with CDK9, building on the crystal structure of CDK9/Flavopiridol (PDB 3BLR) to deduce which sites a linker could be connected to without disrupting key binding interactions. They identified that the 8-position on the flavone scaffold would be ideal for this purpose and, in separate SAR studies on wogonin analogues, found that substituent groups could be introduced at this site without loss of potency. Synthesis of an array of PROTACs with different linkers yielded PROTAC 98, which maintained inhibition of CDK9 (IC_50_ = 520 nM) and exerted an antiproliferative effect on MCF-7 (breast) and L02 (liver) cancer cell lines (Figure 21). The docking pose of 98 in complex with the CDK9 active site supported that the key binding interactions of wogonin were preserved. Computational modelling of the TC is another approach that could be invaluable in structure-based rational PROTAC design, especially since co-crystal structures are not available in most cases. Drummond and Williams [143], described four *in silico* methods for modelling of the TC: 1) sampling the entire TC at once; 2) sampling PROTAC conformations independent of the proteins before adding these in as rigid bodies; 3) sampling the linker conformation in the PROTAC bound to one of the proteins and then adding in the second; and 4) sampling PROTAC conformations independently, but providing possible E3-POI interactions by protein-protein docking. They used each of these methods (along with a series of method-specific filters) to model the TC formed by MZ1 (1) in complex with VHL and BRD4^BD2^ and compared this to the published TC crystal structure (PDB 5T35) [54]. The “hit rate” was defined as the proportion of crystal-like poses with a root-mean-square deviation (RMSD) value within 10 Å of the 5T35 structure. Hit rates varied considerably from 0% (method 1) to 40% (method 4), and the authors acknowledged that determining which poses were crystal-like *a priori* would be difficult, limiting the ability of computational modelling to replace the usage of x-ray crystal structures for the present. Reports have emerged in the literature, however, of using computational TC modelling *a posteriori* to rationalise the SAR of PROTAC analogue libraries obtained via empirical optimisation. Yang et al. [144], synthesised a series of PROTACS for HDAC6 of varying linker lengths using click chemistry, and identified PROTAC NH2 (99) as a potent HDAC6 degrader in MM.1S cells (DC_50_ = 3.2 nM). To investigate intermolecular interactions in the TC, they performed molecular docking studies on the HDAC6-NH2-CRBN complex. The HDAC6-CRBN complex was initially modelled using the Schrödinger protein-protein docking workflow, and the top 100 complex conformations were used to dock NH2 at the PPI interface with the PROTAC occupying the binding site of both protein partners. This suggested several possible TC structures driving H-bond interactions between the triazole of NH2 and Tyr151 from CRBN, and between several surface exposed residues on HDAC6 and CRBN. Docking of analogous and equipotent (DC_50_ = 3.8 nM in MM.1S cells) PROTAC NP8 (100) [145], which possessed an alternate linker conjugation site, highlighted a different set of interactions stabilising the HDAC6-NP8-CRBN TC (Figure 21): a H-bond interaction between the triazole moiety of 100 and Lys157 of CRBN; a H-bond between the urea group in 100 and Ser531 in HDAC6; and a different assortment of PPIs. These results demonstrated that PROTACs with different linker conjugation sites could still form productive TCs stabilised by different interactions, and thus achieve equivalent degradation potency. Similarly, Wang et al. [146], identified degraders that were selective for MCL-1 (101, DC_50_ = 0.7 μM) and BCL-2 (102, DC_50_ = 3.0 μM) in HeLa cells, despite containing the same pan selective naphthalimide based warhead binding to both POIs with similar low micromolar affinities (Figure 21). To rationalise this selectivity, they docked 101 and 102 in the previously published structures of MCL-1/BLC-2 (PDB 2PQK/2XAO) and CRBN (PDB 4TZ4) and used molecular dynamics simulations to relax each structure into a low energy conformation. This suggested that the alkyl linker of 101 engages in hydrophobic interactions with His252 in MCL-1 and folds to enable the α3 helix of MCL1 to come in contact with CRBN, whilst the PEG linker of 102 adopts a linear conformation that brings the α4 helix of BCL-2 near CRBN; numerous PPIs stabilise the TC in each case. PROTAC 101 did not show any favourable conformations in its modelled TC with BCL-2, and vice versa.

**Figure 22.**
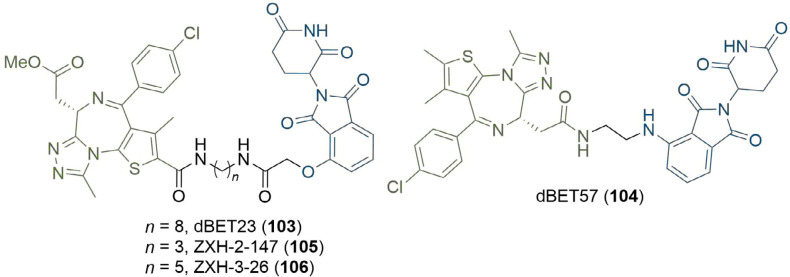
Rational PROTAC design using crystal structures and computational methods. The crystal structures of TC bound 103 and 104, alongside protein-protein docking, revealed potential binding orientations in the TC. Docking was used to identify the shortest distance that could bridge BRD4 and CRBN in the TC, and was used to design 105 and 106

Nowak et al. [147], used protein-protein docking in combination with x-ray crystal structures to rationally design a BRD4-selective degrader. They first generated a library of degraders conjugating JQ1 (58) to thalidomide (9), and solved the crystal structures of their TC with the thalidomide binding domain of CRL4^CRBN^ and BRD4^BD1^. Degraders with linkers of a similar length to representative PROTAC dBET23 (103) induced comparable TC architectures, but PROTACs with shorter linkers, such as dBET57 (104) produced TCs involving distinct PPI surfaces (Figure 22). This was not completely unexpected, since dBET57 contains a two carbon linker, while a minimum of approximately eight carbons would be required to bridge the distance between the E3 and POI binding sites in the dBET23 crystal structure (PDB 6BN7), and is also conjugated from a different position on the warhead. The crystal structure of dBET57 (PDB 6BNB) in complex with CRL4^CRBN^ and BRD4^BD1^ had limited resolution (6.3 Å) but was sufficient to determine that BRD4^BD1^ interacts with the *C*-terminal domain of CRBN, and so recruits a different set of residues for PPIs than dBET23. This led to the hypothesis that CRBN and BRD4 can bind in multiple relative orientations depending on the recruiting PROTAC, which was investigated further using *in silico* protein-protein docking. The crystal structures of lenalidomide (11) in complex with CRBN (PDB 4TZ4) and JQ1 in complex with BRD4^BD1^ (PDB 3MXF) were used to perform a global protein docking experiment leading to 20, 000 structural models. In the absence of a PROTAC molecule both proteins afforded a wide range of energy minima, out of which a conformation closely resembling the TC obtained with dBET23 (103) could be identified among the top 200 conformations. To test whether this information could be used in the rational design of next generation degraders, the authors calculated the shortest distances between solvent exposed regions of JQ1 and lenalidomide in their top 200 poses, and found that a distance of 3-4 Å (corresponding to a linker length of 2-3 atoms) would be sufficient to bridge the gap. This informed the design of ZXH-2-147 (105), where the carbon linker in 103 was shortened by five carbons. PROTAC 105 displayed degradation activity against BRD4^BD1^, but extension of the linker by two carbons was required to yield the potent (DC_50_ = 5 nM in HEK293T cells) and isoform-selective (no BRD2/3 degradation at >10 μM) PROTAC ZXH-3-26 (106). They reasoned that the greater selectivity obtained with a shorter linker could be due to the large reduction in the number of accessible binding conformations; the binding conformation of the PROTAC in a TC with a particular POI may not be accessible in structurally related proteins. This work is an important example of computational modelling providing an alternative to x-ray crystallography in rational design; modelling of PPIs in the absence of a linker provided structural information that could be used to inform on likely lengths and vectors for optimal degradation.

**Figure 23.**
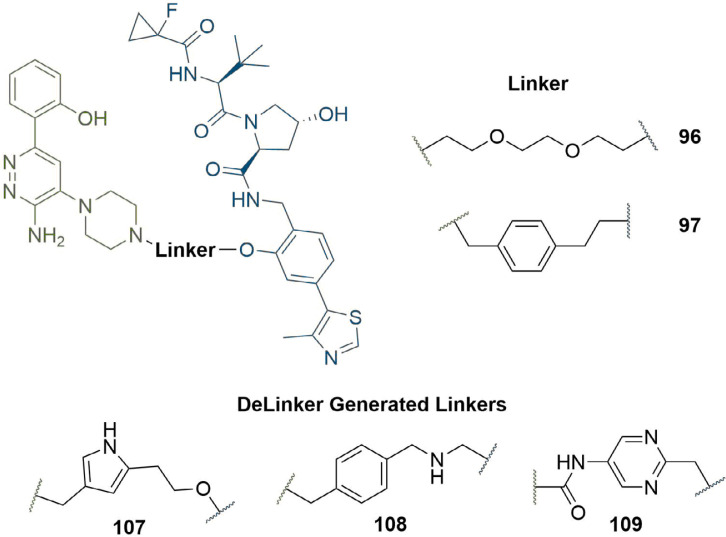
The generative model DeLinker was used to generate novel PROTAC linkers 107-109 to attain the same objective as 97 in improving the interactions of 96 in the TC

A possible alternate to the aforementioned methods for structure-based rational design of PROTAC molecules could be in the harnessing of machine learning models. Imrie et al. [148], created a graph-based deep generative model for the design of molecules incorporating two separate fragments. In essence, their DeLinker method assesses the relative spatial position and orientation of two fragment molecules, and incorporates both into one molecule by either generating or replacing the linker between them. This is achieved via an iterative “bond by bond” process whereby new atoms are incorporated one at a time from a set of permitted atom types. The features of this model design would seem to lend themselves well to the requirements of PROTAC design: the structures of the two fragments are retained; linkage only occurs from specified exit vectors; and the length of the linker the model will generate can be specified. The authors tested DeLinker on the model SMARCA-degrading PROTACs reported by the Ciulli group [80]. As previously described, Farnaby et al. [80], sought to optimise the interaction of their linker in 96 to a hydrophobic region created by Tyr98 in VHL, and achieved this by introduction of a 1,4-disubstituted phenyl ring to produce 97. Imrie et al. [148], investigated whether their model could design alternate linkers to the PEG chain in 96 that could retain the same interactions and conformation. They first generated conformers of the anchor/warhead that constrained them to poses close to their reported binding conformations (PDB 6HAY), and then used DeLinker to produce over 2, 000 unique structures where these were linked together into PROTACs. Of these generated structures, three contained aromatic linkers (none of which were in the training set) that could closely recreate the same linker conformation as observed in 96 (PROTACs 107–109, Figure 23). When these structures were minimised in docking experiments they each scored equivalent to or better than 97, and substantially better than 96, although this was not validated experimentally. This work demonstrates the potential for machine learning to be a useful addition to the toolkit for rational PROTAC design in the future.

## Conclusion and outlook

The PROTAC technology has established itself as a promising strategy to address challenging POI targets that have proved recalcitrant to classical occupancy-based or targeted covalent inhibitors. In this review, we highlight that while synthetically tractable alkyl and PEG linkers have dominated PROTAC design for more than a decade, the PROTAC field is currently undergoing a paradigm shift towards more sophisticated and rationally designed functional linkers. Accumulating experimental evidence in the last decade has pointed at the important relationship between the overall degradation efficiency, selectivity, and properties of PROTACs and the characteristics of its linker, such as length, chemical composition, and the site and vector of conjugation. Identifying suitable exit vectors for the anchor/warhead can be relatively straightforward and informed by visual inspection of their individual bound crystal structures or docking poses, in instances where these structures are available. Comparatively, designing PROTACs with suitable linkers is a relatively labour intensive and empirical exercise. In proof of concept studies, PROTACs with linkers sampling a range of lengths are usually synthesised. This allows for probing of the spatial and conformational ensemble of the TC, so that productive conformations which will allow the successful ubiquitination of the POI can be accessed. The distance between the POI and Ub/E2, their relative orientation, and the presentation and accessibility of suitably reactive POI lysine residues to the E2, are important properties which ultimately depend on the linker unit. However, the relationship between the spatial distribution of lysine residues at the POI surface, the architecture and connectivity of the poly-Ub chains, and the overall efficiency of degradation are still poorly understood. The complexity of these ensembles, along with their associated energy landscapes, makes it extremely challenging to predict *a priori* which combination of anchor, linker, and warhead will lead to optimal degradation.

An important caveat in the field is the lack of reliable and general methods to study the structure of POI/E3 assemblies, and rationalise experimental observations. As a notable example, while high resolution crystal structures of the eight individual BET bromodomains have been solved, the structures of the four full-length proteins have remained elusive. Recent reports of *in vitro*/*in silico* functional and structural studies of TCs have highlighted useful approaches and models to attempt rationalising cellular SAR data. However, it will be interesting to determine whether *in vitro* biophysical/biochemical binding studies in addition to investigating TC structures by X-ray crystallography or computational modeling using truncated constructs (e.g., BET bromodomains) are sufficiently representative of their full length cellular counterparts for making accurate predictions of the cellular activity and selectivity profiles of PROTACs. A systematic *in vitro* assessment of binding properties, notably cooperativity, and TC structure will be critical to bridge this gap. The ever increasing sensitivity of structural techniques will likely play a pivotal role in shedding light on the structure of a wide range of relevant POIs and their TCs with diverse E3 ligases, in turn providing crucial insight into the impact of the linker on TC formation. While still technically/financially demanding, cryo-electron microscopy (cryo-EM) seems well positioned in this respect, notably due to its solution phase nature and suitability for studying high MW (usually > 100 kDa) assemblies. Critically, recent reports of prototypical cryo-EM structures at atomic resolution, along with fragment bound protein structures suggest that the gap in resolution between X-ray crystallography and cryo-EM is closing quickly [149–151]. NMR is another technique likely to find utility here: conformational analysis of PROTACs in solution using NMR methods has been reported [152], and could further provide information on the bound PROTAC conformation in the TC [153]. Further, ligand observed NMR experiments such as saturation transfer difference and group epitope mapping could be useful in the future for identifying suitable warhead exit vectors where crystal structures are unavailable [154]. Group epitope mapping is a well-established method and has proven reliable in determining solvent exposed positions in a range of VHL ligands [155]. Computational modelling of the TC as an alternative to experimental techniques to inform rational design is also likely to see significant expansion in the future, and an increasing number of groups are developing methods to do this [156, 157]. The continued development of TC modelling may provide avenues for linker development in lieu of significant empirical experimental optimisation. Key considerations such as length, selection of constituent linker motifs, and optimisation of protein-PROTAC interactions in the TC could be addressed with a reduced requirement for the synthesis of a large number of linker structures.

Beyond structural biology, devising bioactive PROTACs is often complicated by issues surrounding their pharmacokinetic properties, notably cell permeability, metabolic stability, and solubility. As a result, the cell activity of a PROTAC is difficult to predict. An increasing number of examples in the literature have highlighted how changes in the composition of the linker away from simple alkyl and PEG units, such as the incorporation of polar rigidifying groups, can significantly improve these properties and optimise a PROTAC’s DMPK profile (refer to Figures 9 and 17) [158]. We expect this trend towards more rigid and polar linkers to continue, especially since the improvement in TC modelling can identify opportunities to incorporate rigidifying moieties whilst retaining the PROTAC’s bioactive conformation. Beyond property optimisation, there are increasing examples of PROTAC linkers employed for a functional purpose, such as incorporating photoswitchable diazobenzene units to enable spatiotemporal control of PROTAC activity, and it will be interesting to see what new advances will be made in this field in the near future. Structurally simple diazobenzenes often suffer from a number of drawbacks limiting their therapeutic potential [159]. These notably include moderate control on *cis*/*trans* ratios at photo-stationary states and the requirement of high-energy UV light for photo-isomerisation, with potential detrimental consequences for on/off target effect and cell/tissue penetration and damage, respectively. These properties will need to be scrutinised closely in the future for the development of clinically viable photo-controlled PROTAC drug candidates. The recent development of prototypical cell active PROTACs such as 79 displaying red-shifted photo-isomerisation wavelengths suggests that it is progressively being addressed. Equally important, light delivery and the suitability of light responsive PROTACs for the development of *in vivo* photodynamic therapies will likely depend on the targeted POI and tissue distribution, and its accessibility by the light source and activation wavelength(s). Even whilst preparing this manuscript, examples of new approaches to linker design and function have been described: an example of combinatorial PROTAC library synthesis using the coupling of an aldehyde to a hydrazide, to quickly screen different linker lengths and compositions [160]; and a scaffold-hopping approach using core changes of the PROTAC structure to arrive at a potent degrader, whilst circumventing the need for lengthy optimisation of the linker [161].

Much work still remains to fully understand and rationalise the development of efficient and general linking strategies, with huge opportunities for increased affinity and/or specificity of the resulting PROTACs. This may for example be achieved by enhancing cooperativity via designing linkers engaging in specific interactions in a unique TC, to provide extra affinity and target specificity. The recent observations that PROTACs are able to induce isoform specific knockdown in certain conditions suggests that it may be achievable [12, 56]. Of note, BRD4 selective degraders represent an attractive and complementary alternative to the “Bump-and-Hole” chemical genetics approach previously reported by us and others for allele selective BET bromodomain inhibition [139, 162, 163]. While not generalizable to the other three BET proteins, PROTAC mediated BRD4 degradation presents an important technical advantage removing the need for extensive protein and ligand engineering. Enhancing TC cooperativity may also help in modifying the well documented “hook effect”, whereby the formation of PROTAC TCs is inhibited at high PROTAC concentrations by the formation of PROTAC-E3/POI binary complexes [164]. A deeper understanding of the stability of the TC will be crucial to achieve this, and may be quantified through techniques such as surface plasmon resonance (SPR), as recently demonstrated by Roy et al [55]. In particular, the researchers observed that TCs with long half-lives displayed enhanced cooperativities and more favourable degradation profiles in comparison to those with fast dissociation kinetics, a finding which has subsequently been validated by Pillow et al [165]. The use of biophysical techniques to monitor TC kinetics when optimising PROTAC linker chemistry may become more commonplace in future as a strategy to improve PROTAC potency and selectivity whilst mitigating the hook effect. As discussed previously, the ability to monitor and enhance TC stability and cooperativity will facilitate construction of PROTACs based on weaker affinity warheads [59].

It now remains to be seen if the lessons learnt from these examples can allow derivation of general design principles for the development of PROTAC degraders targeting specific isoforms across protein families sharing high structural homology at their active site. This is a recurrent challenge for a number of protein families, including kinases and epigenetic effectors. More generally, it is also worth noting that the molecular basis underlying the differing selectivity profiles of certain PROTACs is far from being fully elucidated. For example, it is not clear whether differing degradation selectivity profiles of reported BET degraders directly result from the varying PROTAC structures (refer to Figure 2), or whether the genetic, epigenetic and proteomic backgrounds of different cell lines also impact the observed potency and selectivity, and to what extent. The cellular abundance of a particular ligase, relative cellular levels of multiple POIs and their dynamic post-translational modification, localization and involvement in high affinity interactions with cellular partners (e.g., histone tails), along with the composition of the various structural elements delineating cellular compartments can present significant variability across different cell lines. These factors may have an impact on the accessibility of a given POI to the PROTAC, but are not systematically evaluated in the wider PROTAC literature. As a result, it is not always clear which primary cellular models are most appropriate to assess the bioactivity of a given PROTAC, and its potential for future translational studies. This is another important pinch-point which we believe will require more consideration in the future. In particular, it is unclear whether post-translational modifications of the POI can impact the potency of a PROTAC, and whether this may be harnessed to target specific proteins sub-populations in a posttranslational modification (PTM)-dependant manner. While there is limited room for manoeuvre to alter the anchor and warhead, synthetic modification of the linker towards designer properties and functions will represent the next frontier to overcome these obstacles.

## Abbreviations

Ad: adapter protein
AR: androgen receptor
BD: bromodomain
BET: Bromo- and Extra-Terminal
BTK: Bruton’s tyrosine kinase
C481S: Cys481 to Ser
cIAP: cellular inhibitor of apoptosis protein
CRBN: cereblon
cryo-EM: cryo-electron microscopy
Deg_S: degrader score
DIPEA: *N,N*-diisopropylethylamine
DMNB: 4,5-dimethoxy-2-nitrobenzyl
DMPK: drug metabolism and pharmacokinetic
DSG: di(*N*-succinimidyl) glutarate
DSS: di(*N*-succinimidyl) suberate
ER: oestrogen receptor
ERK: extracellular signal-regulated kinase
HBD: hydrogen-bond donor
HDAC: histone deacetylase
HER2: human epidermal growth factor receptor 2
HIF-1α: hypoxia-inducible factor 1α
MDM2: murine double minute 2
MetAP-2: mammalian methionine aminopeptidase type 2
MW: molecular weight
PDB: protein database
PDP: protein degradation probe
POI: protein of interest
PPIs: protein-protein interactions
PROTAC: proteolysis targeting chimeras
PSS: photostationary states
SAR: structure-activity relationship
SBD: substrate binding domain
Sc: scaffolding protein
SCF: Skp1-Cullin-F box complex
SGK3: serum- and glucocorticoid-regulated kinase family member 3
S_N_Ar: nucleophilic aromatic substitution
SNIPER: specific and nongenetic IAP-dependent protein erasers
TC: ternary complex
TCO: *trans*-cyclooctene
TPSA: topological polar surface area
Ub: ubiquitin
UPS: ubiquitin-proteasome system
VCB: VHL-ElonginC-ElonginB
VHL: Von Hippel-Lindau tumour suppressor protein

## Declarations

### Copyright

© The Author(s) 2020.

## References

[1] Paiva SL Crews CM. Targeted protein degradation: elements of PROTAC design. Curr Opin Chem Biol. 2019;50:111–9. 10.1016/j.cbpa.2019.02.022 31004963 PMC6930012

[2] Sun X Gao H Yang Y He M Wu Y Song Y PROTACs: great opportunities for academia and industry. Signal Transduction Targeted Ther. 2019;4:64. 10.1038/s41392-019-0101-6 PMC6927964 31885879

[3] Burslem GM Crews CM. Small-molecule modulation of protein homeostasis. Chem Rev. 2017;117:11269–301. 10.1021/acs.chemrev.7b00077 28777566

[4] Huang X Dixit VM. Drugging the undruggables: exploring the ubiquitin system for drug development. Cell Res. 2016;26:484–98. 10.1038/cr.2016.31 27002218 PMC4822129

[5] Schapira M Calabrese MF Bullock AN Crews CM. Targeted protein degradation: expanding the toolbox. Nat Rev Drug Discov. 2019;18:949–63. 10.1038/s41573-019-0047-y 31666732

[6] Schneekloth JS Jr Fonseca FN Koldobskiy M Mandal A Deshaies R Sakamoto K Chemical genetic control of protein levels: selective *in vivo* targeted degradation. J Am Chem Soc. 2004;126:3748–54. 10.1021/ja039025z 15038727

[7] Adams J. The proteasome: structure, function, and role in the cell. Cancer Treat Rev. 2003;29 Suppl 1:3–9. 10.1016/S0305-7372(03)00081-1 12738238

[8] Metzger MB Hristova VA Weissman AM. HECT and RING finger families of E3 ubiquitin ligases at a glance. J Cell Sci. 2012;125:531–7. 10.1242/jcs.091777 22389392 PMC3381717

[9] Deshaies RJ Joazeiro CA. RING domain E3 ubiquitin ligases. Annu Rev Biochem. 2009;78:399–434. 10.1146/annurev.biochem.78.101807.093809 19489725

[10] Zheng N Shabek N. Ubiquitin ligases: structure, function, and regulation. Annu Rev Biochem. 2017;86:129–57. 10.1146/annurev-biochem-060815-014922 28375744

[11] Bondeson DP Mares A Smith IE Ko E Campos S Miah AH Catalytic *in vivo* protein knockdown by small-molecule PROTACs. Nat Chem Biol. 2015;11:611–7. 10.1038/nchembio.1858 26075522 PMC4629852

[12] Zengerle M Chan KH Ciulli A. Selective small molecule induced degradation of the BET bromodomain protein BRD4. ACS Chem Biol. 2015;10:1770–7. 10.1021/acschembio.5b00216 26035625 PMC4548256

[13] Crews CM. Targeting the undruggable proteome: the small molecules of my dreams. Chem Biol. 2010;17:551–5. 10.1016/j.chembiol.2010.05.011 20609404 PMC2925121

[14] Zhou H Bai L Xu R Zhao Y Chen J McEachern D Structure-based discovery of SD-36 as a potent, selective, and efficacious PROTAC degrader of STAT3 protein. J Med Chem. 2019;62:11280–300. 10.1021/acs.jmedchem.9b01530 31747516 PMC8848307

[15] Lu J Qian Y Altieri M Dong H Wang J Raina K Hijacking the E3 ubiquitin ligase cereblon to efficiently target BRD4. Chem Biol. 2015;22:755–63. 10.1016/j.chembiol.2015.05.009 26051217 PMC4475452

[16] Winter GE Buckley DL Paulk J Roberts JM Souza A Dhe-Paganon S Phthalimide conjugation as a strategy for *in vivo* target protein degradation. Science. 2015;348:1376–81. 10.1126/science.aab1433 25999370 PMC4937790

[17] Sakamoto KM Kim KB Kumagi A Mercurio F Crews CM Deshaies RJ. Protacs: chimeric molecules that target proteins to the Skp1-Cullin-F box complex for ubiquitination and degradation. Proc Natl Acad Sci U S A. 2001;98:8554–9. 10.1073/pnas.141230798 11438690 PMC37474

[18] Sakamoto KM Kim KB Verma R Ransick A Stein B Crews CM Development of Protacs to target cancer-promoting proteins for ubiquitination and degradation. Mol Cell Proteomics. 2003;2:1350–8. 10.1074/mcp.T300009-MCP200 14525958

[19] Fosgerau K Hoffmann T. Peptide therapeutics: current status and future directions. Drug Discov Today. 2015;20:122–8. 10.1016/j.drudis.2014.10.003 25450771

[20] Schneekloth AR Pucheault M Tae HS Crews CM. Targeted intracellular protein degradation induced by a small molecule: en route to chemical proteomics. Bioorg Med Chem Lett. 2008;18:5904–8. 10.1016/j.bmcl.2008.07.114 18752944 PMC3175619

[21] Hines J Lartigue S Dong H Qian Y Crews CM. MDM2-recruiting PROTAC offers superior, synergistic antiproliferative activity via simultaneous degradation of BRD4 and stabilization of p53. Cancer Res. 2019;79:251–62. 10.1158/0008-5472.CAN-18-2918 30385614 PMC6318015

[22] Bulatov E Ciulli A. Targeting Cullin-RING E3 ubiquitin ligases for drug discovery: structure, assembly and small-molecule modulation. Biochem J. 2015;467:365–86. 10.1042/BJ20141450 25886174 PMC4403949

[23] Galdeano C Gadd MS Soares P Scaffidi S Van Molle I Birced I Structure-guided design and optimization of small molecules targeting the protein-protein interaction between the von Hippel– Lindau (VHL) E3 ubiquitin ligase and the hypoxia inducible factor (HIF) alpha subunit with *in vitro* nanomolar affinities. J Med Chem. 2014;57:8657–63. 10.1021/jm5011258 25166285 PMC4207132

[24] Buckley DL Gustafson JL Van Molle I Roth AG Tae HS Gareiss PC Small-molecule inhibitors of the interaction between the E3 ligase VHL and HIF1α. Angew Chem Int Ed Engl. 2012;51:11463–7. 10.1002/anie.201206231 23065727 PMC3519281

[25] Semenza GL. Life with oxygen. Science. 2007;318:62–4. 10.1126/science.1147949 17916722

[26] Hon WC Wilson MI Harlos K Claridge TDW Schofield CJ Pugh CW Structural basis for the recognition of hydroxyproline in HIF-1α by pVHL. Nature. 2002;417:975–8. 10.1038/nature00767 12050673

[27] Brahimi-Horn MC Pouysségur J. Harnessing the hypoxia-inducible factor in cancer and ischemic disease. Biochem Pharmacol. 2007;73:450–7. 10.1016/j.bcp.2006.10.013 17101119

[28] Clague MJ Heride C Urbé S. The demographics of the ubiquitin system. Trends Cell Biol. 2015;25:417–26. 10.1016/j.tcb.2015.03.002 25906909

[29] Brand M Winter GE. Stick it to E3s. Nat Chem Biol. 2019;15:655–6. 10.1038/s41589-019-0312-8 31209352

[30] Lai AC Toure M Hellerschmied D Salami J Jamie-Figueroa S Ko E Modular PROTAC design for the degradation of oncogenic BCR-ABL. Angew Chem Int Ed Engl. 2016;55:807–10. 10.1002/anie.201507634 26593377 PMC4733637

[31] Zhang L Riley-Gillis B Vijay P Shen Y. Acquired resistance to BET-PROTACs (proteolysis-targeting chimeras) caused by genomic alterations in core components of E3 ligase complexes. Mol Cancer Ther. 2019;18:1302–11. 10.1158/1535-7163.MCT-18-1129 31064868

[32] Naito M Ohoka N Shibata N. SNIPERs–Hijacking IAP activity to induce protein degradation. Drug Discov Today Technol. 2019;31:35–42. 10.1016/j.ddtec.2018.12.002 31200857

[33] Itoh Y Kitaguchi R Ishikawa M Naito M Hashimoto Y. Design, synthesis and biological evaluation of nuclear receptor-degradation inducers. Bioorg Med Chem. 2011;19:6768–78. 10.1016/j.bmc.2011.09.041 22014751

[34] Itoh Y Ishikawa M Naito M Hashimoto Y. Protein knockdown using methyl bestatin-ligand hybrid molecules: design and synthesis of inducers of ubiquitination-mediated degradation of cellular retinoic acid-binding proteins. J Am Chem Soc. 2010;132:5820–6. 10.1021/ja100691p 20369832

[35] Ohoka N Okuhira K Ito M Nagai K Shibata N Hattori T *In vivo* knockdown of pathogenic proteins via specific and nongenetic inhibitor of apoptosis protein (IAP)-dependent protein erasers (SNIPERs). J Biol Chem. 2017;292:4556–70. 10.1074/jbc.M116.768853 28154167 PMC5377772

[36] Ohoka N Morita Y Nagai K Shimokawa K Ujikawa O Fujimori I Derivatization of inhibitor of apoptosis protein (IAP) ligands yields improved inducers of estrogen receptor α degradation. J Biol Chem. 2018;293:6776–90. 10.1074/jbc.RA117.001091 29545311 PMC5936811

[37] Zhang X Crowley VM Wucherpfennig TG Dix MM Cravatt BF. Electrophilic PROTACs that degrade nuclear proteins by engaging DCAF16. Nat Chem Biol. 2019;15:737–46. 10.1038/s41589-019-0279-5 31209349 PMC6592777

[38] Ward CC Kleinman JI Brittain SM Lee PS Chung CYS Kim K Covalent ligand screening uncovers a RNF4 E3 ligase recruiter for targeted protein degradation applications. ACS Chem Biol. 2019;14:2430–40. 31059647 10.1021/acschembio.8b01083 PMC7422721

[39] Lu M Liu T Jiao Q Ji J Tao M Liu Y Discovery of a Keap1-dependent peptide PROTAC to knockdown Tau by ubiquitination-proteasome degradation pathway. Eur J Med Chem. 2018;146:251–9. 10.1016/j.ejmech.2018.01.063 29407955

[40] Ottis P Toure M Cromm PM Ko E Gustafson JL Crews CM. Assessing different E3 ligases for small molecule induced protein ubiquitination and degradation. ACS Chem Biol. 2017;12:2570–8. 10.1021/acschembio.7b00485 28767222

[41] Nunes J McGonagle GA Eden J Kiritharan G Touzet M Lewell X Targeting IRAK4 for degradation with PROTACs. ACS Med Chem Lett. 2019;10:1081–5. 10.1021/acsmedchemlett.9b00219 31312412 PMC6627720

[42] de Wispelaere M Du G Donovan KA Zhang T Eleuteri NA Yuan JC Small molecule degraders of the hepatitis C virus protease reduce susceptibility to resistance mutations. Nat Commun. 2019;10:3468. 10.1038/s41467-019-11429-w 31371704 PMC6672008

[43] Silva MC Ferguson FM Cai Q Donovan KA Nandi G Patnaik D Targeted degradation of aberrant tau in frontotemporal dementia patient-derived neuronal cell models. eLife. 2019;8:e45457. 10.7554/eLife.45457 30907729 PMC6450673

[44] Chu TT Gao N Li QQ Chen PG Yang XF Chen YX Specific knockdown of endogenous Tau protein by peptide-directed ubiquitin-proteasome degradation. Cell Chem Biol. 2016;23:453–61. 10.1016/j.chembiol.2016.02.016 27105281

[45] Lee H Puppala D Choi EY Swanson H Kim KB. Targeted degradation of the aryl hydrocarbon receptor by the PROTAC approach: a useful chemical genetic tool. ChemBioChem. 2007;8:2058–62. 10.1002/cbic.200700438 17907127

[46] Zhao B Burgess K. PROTACs suppression of CDK4/6, crucial kinases for cell cycle regulation in cancer. Chem Commun (Camb). 2019;55:2704–7. 10.1039/c9cc00163h 30758029

[47] Burslem GM Smith BE Lai AC Jaime-Figueroa S McQuaid DC Bondeson DP The advantages of targeted protein degradation over inhibition: an RTK case study. Cell Chem Biol. 2018;25:67–77.e3. 10.1016/j.chembiol.2017.09.009 29129716 PMC5831399

[48] Zoppi V Hughes SJ Maniaci C Testa A Gmaschitz T Wieshofer C Iterative design and optimization of initially inactive proteolysis targeting chimeras (PROTACs) identify VZ185 as a potent, fast, and selective von Hippel-Lindau (VHL) based dual degrader probe of BRD9 and BRD7. J Med Chem. 2019;62:699–726. 10.1021/acs.jmedchem.8b01413 30540463 PMC6348446

[49] Yang K Song Y Xie H Wu H Wu YT Leisten ED Development of the first small molecule histone deacetylase 6 (HDAC6) degraders. Bioorg Med Chem Lett. 2018;28:2493–7. 10.1016/j.bmcl.2018.05.057 29871848

[50] Honigberg LA Smith AM Sirisawad M Verner E Loury D Chang B The Bruton tyrosine kinase inhibitor PCI-32765 blocks B-cell activation and is efficacious in models of autoimmune disease and B-cell malignancy. Proc Natl Acad Sci U S A. 2010;107:13075–80. 10.1073/pnas.1004594107 20615965 PMC2919935

[51] Gabizon R Shraga A Gehrtz P Livnah E Shorer Y Gurwicz N Efficient targeted degradation via reversible and irreversible covalent PROTACs. J Am Chem Soc. 2020;142:11734–42. 10.1021/jacs.9b13907 32369353 PMC7349657

[52] Arthur R Valle-Argos B Steele AJ Packham G. Development of PROTACs to address clinical limitations associated with BTK-targeted kinase inhibitors. Explor Target Antitumor Ther. 2020;1:131–52. 10.37349/etat.2020.00009 32924028 PMC7116064

[53] Sun Y Zhao X Ding N Gao H Wu Y Yang Y PROTAC-induced BTK degradation as a novel therapy for mutated BTK C481S induced ibrutinib-resistant B-cell malignancies. Cell Res. 2018;28:779–81. 10.1038/s41422-018-0055-1 29875397 PMC6028582

[54] Gadd MS Testa A Lucas X Chan KH Chen W Lamont DJ Structural basis of PROTAC cooperative recognition for selective protein degradation. Nat Chem Biol. 2017;13:514–21. 10.1038/nchembio.2329 28288108 PMC5392356

[55] Bondeson DP Smith BE Burslem GM Buhimschi AD Hines J Jaime-Figueroa S Lessons in PROTAC design from selective degradation with a promiscuous warhead. Cell Chem Biol. 2018;25:78–87.e5. 10.1016/j.chembiol.2017.09.010 29129718 PMC5777153

[56] Smith BE Wang SL Jaime-Figueroa S Harbin A Wang J Hamman BD Differential PROTAC substrate specificity dictated by orientation of recruited E3 ligase. Nat Commun. 2019;10:131. 10.1038/s41467-018-08027-7 30631068 PMC6328587

[57] Mullard A. First targeted protein degrader hits the clinic. Nat Rev Drug Discov. 2019;18:237–9. 10.1038/d41573-019-00043-6 30936511

[58] Liu J Ma J Liu Y Xia J Li Y Wang ZP PROTACs: a novel strategy for cancer therapy. Semin Cancer Biol. 2020;[Epub ahead of print]. 10.1016/j.semcancer.2020.02.006 32058059

[59] Roy MJ Winkler S Hughes SJ Whitworth C Galant M Farnaby W SPR-measured dissociation kinetics of PROTAC ternary complexes influence target degradation rate. ACS Chem Biol. 2019;14:361–8. 10.1021/acschembio.9b00092 30721025 PMC6423499

[60] Zorba A Nguyen C Xu Y Starr J Borzilleri K Smith J Delineating the role of cooperativity in the design of potent PROTACs for BTK. Proc Natl Acad Sci U S A. 2018;115:E7285–92. 10.1073/pnas.1803662115 30012605 PMC6077745

[61] Borsari C Trader DJ Tait A Costi MP. Designing chimeric molecules for drug discovery by leveraging chemical biology. J Med Chem. 2020;63:1908–28. 10.1021/acs.jmedchem.9b01456 32023055 PMC7997565

[62] Maple HJ Clayden N Baron A Stacey C Felix R. Developing degraders: principles and perspectives on design and chemical space. MedChemComm. 2019;10:1755–64. 10.1039/c9md00272c 31867093 PMC6894040

[63] Smalley JP Adams GE Millard CJ Song Y Norris JKS Schwabe JWR PROTAC-mediated degradation of class I histone deacetylase enzymes in corepressor complexes. Chem Commun (Camb). 2020;56:4476–9. 10.1039/d0cc01485k 32201871 PMC7610821

[64] Zhang X Xu F Tong L Zhang T Xie H Lu X Design and synthesis of selective degraders of EGFR^L858R/T790M^ mutant. Eur J Med Chem. 2020;192:112199. 10.1016/j.ejmech.2020.112199 32171162

[65] Cyrus K Wehenkel M Choi EY Han HJ Lee H Swanson H Impact of linker length on the activity of PROTACs. Mol Biosyst. 2011;7:359–64. 10.1039/c0mb00074d 20922213 PMC3835402

[66] Zhang D Baek SH Ho A Kim K. Degradation of target protein in living cells by small-molecule proteolysis inducer. Bioorg Med Chem Lett. 2004;14:645–8. 10.1016/j.bmcl.2003.11.042 14741260

[67] Cyrus K Wehenkel M Choi EY Lee H Swanson H Kim KB. Jostling for position: optimizing linker location in the design of estrogen receptor-targeting PROTACS. ChemMedChem. 2010;5:979–85. 10.1002/cmdc.201000146 20512796 PMC3516907

[68] Rana S Bendjennat M Kour S King HM Kizhake S Zahid M Selective degradation of CDK6 by a palbociclib based PROTAC. Bioorg Med Chem Lett. 2019;29:1375–9. 10.1016/j.bmcl.2019.03.035 30935795 PMC6487213

[69] Wang B Wu S Liu J Yang K Xie H Tang W. Development of selective small molecule MDM2 degraders based on nutlin. Eur J Med Chem. 2019;176:476–91. 10.1016/j.ejmech.2019.05.046 31128449

[70] Li Y Yang J Aguilar A McEachern D Przybranowski S Liu L Discovery of MD-224 as a first-in-class, highly potent, and efficacious proteolysis targeting chimera murine double minute 2 degrader capable of achieving complete and durable tumor regression. J Med Chem. 2019;62:448–66. 10.1021/acs.jmedchem.8b00909 30525597 PMC6545112

[71] Yang J Li Y Aguilar A Liu Z Yang CY Wang S. Simple structural modifications converting a bona fide MDM2 PROTAC degrader into a molecular glue molecule: a cautionary tale in the design of PROTAC degraders. J Med Chem. 2019;62:9471–87. 10.1021/acs.jmedchem.9b00846 31560543 PMC7354697

[72] Crew AP Raina K Dong H Qian Y Wang J Vigil D Identification and characterization of von Hippel-Lindau-recruiting proteolysis targeting chimeras (PROTACs) of TANK-binding kinase 1. J Med Chem. 2018;61:583–98. 10.1021/acs.jmedchem.7b00635 28692295

[73] Kim K Lee DH Park S Jo SH Ku B Park SG Disordered region of cereblon is required for efficient degradation by proteolysis-targeting chimera. Sci Rep. 2019;9:19654. 10.1038/s41598-019-56177-5 31873151 PMC6928225

[74] Steinebach C Sosič I Lindner S Bricelj A Kohl F Ng YLD A MedChem toolbox for cereblon-directed PROTACs. MedChemComm. 2019;10:1037–41. 10.1039/c9md00185a 31304001 PMC6596386

[75] Steinebach C Kehm H Lindner S Vu LP Köpff S Mármol MÁL PROTAC-mediated crosstalk between E3 ligases. Chem Commun (Camb). 2019;55:1821–4. 10.1039/c8cc09541h 30672516

[76] Qiu X Sun N Kong Y Li Y Yang X Jiang B. Chemoselective synthesis of lenalidomide-based PROTAC library using alkylation reaction. Org Lett. 2019;21:3838–41. 10.1021/acs.orglett.9b01326 31066567

[77] Qin C Hu Y Zhou B Fernandez-Salas E Yang CY Liu L Discovery of QCA570 as an exceptionally potent and efficacious proteolysis targeting chimera (PROTAC) degrader of the bromodomain and extra-terminal (BET) proteins capable of inducing complete and durable tumor regression. J Med Chem. 2018;61:6685–704. 10.1021/acs.jmedchem.8b00506 30019901 PMC6545111

[78] Han X Wang C Qin C Xiang W Fernandez-Salas E Yang CY Discovery of ARD-69 as a highly potent proteolysis targeting chimera (PROTAC) degrader of androgen receptor (AR) for the treatment of prostate cancer. J Med Chem. 2019;62:941–64. 10.1021/acs.jmedchem.8b01631 30629437

[79] Han X Zhao L Xiang W Qin C Miao B Xu T Discovery of highly potent and efficient PROTAC degraders of androgen receptor (AR) by employing weak binding affinity VHL E3 ligase ligands. J Med Chem. 2019;62:11218–31. 10.1021/acs.jmedchem.9b01393 31804827

[80] Farnaby W Koegl M Roy MJ Whitworth C Diers E Trainor N BAF complex vulnerabilities in cancer demonstrated via structure-based PROTAC design. Nat Chem Biol. 2019;15:672–80. 10.1038/s41589-019-0294-6 31178587 PMC6600871

[81] Shibata N Nagai K Morita Y Ujikawa O Ohoka N Hattori T Development of protein degradation inducers of androgen receptor by conjugation of androgen receptor ligands and inhibitor of apoptosis protein ligands. J Med Chem. 2018;61:543–75. 10.1021/acs.jmedchem.7b00168 28594553

[82] Xia LW Ba MY Liu W Cheng W Hu CP Zhao Q Triazol: a privileged scaffold for proteolysis targeting chimeras. Future Med Chem. 2019;11:2919–73. 10.4155/fmc-2019-0159 31702389

[83] Kolb HC Finn MG Sharpless KB. Click chemistry: diverse chemical function from a few good reactions. Angew Chem Int Ed Engl. 2001;40:2004–21. 10.1002/1521-3773(20010601)40:11<2004::aid-anie2004>3.3.co;2-x 11433435

[84] Moses JE Moorhouse AD. The growing applications of click chemistry. Chem Soc Rev. 2007;36:1249–62. 10.1039/b613014n 17619685

[85] Chen H Chen F Liu N Wang X Gou S. Chemically induced degradation of CK2 by proteolysis targeting chimeras based on a ubiquitin–proteasome pathway. Bioorg Chem. 2018;81:536–44. 10.1016/j.bioorg.2018.09.005 30245235

[86] Zhou L Chen W Cao C Shi Y Ye W Hu J Design and synthesis of α-naphthoflavone chimera derivatives able to eliminate cytochrome P450 (CYP)1B1-mediated drug resistance via targeted CYP1B1 degradation. Eur J Med Chem. 2020;189:112028. 10.1016/j.ejmech.2019.112028 31945665

[87] Wurz RP Dellamaggiore K Dou H Javier N Lo MC McCarter JD A “click chemistry platform” for the rapid synthesis of bispecific molecules for inducing protein degradation. J Med Chem. 2018;61:453–61. 10.1021/acs.jmedchem.6b01781 28378579

[88] Zhao Q Lan T Su S Rao Y. Induction of apoptosis in MDA-MB-231 breast cancer cells by a PARP1-targeting PROTAC small molecule. Chem Commun (Camb). 2019;55:369–72. 10.1039/c8cc07813k 30540295

[89] Schiedel M Herp D Hammelmann S Swyter S Lehotzky A Robaa D Chemically induced degradation of sirtuin 2 (Sirt2) by a proteolysis targeting chimera (PROTAC) based on sirtuin rearranging ligands (SirReals). J Med Chem. 2018;61:482–91. 10.1021/acs.jmedchem.6b01872 28379698

[90] Lebraud H Wright DJ Johnson CN Heightman TD. Protein degradation by in-cell self-assembly of proteolysis targeting chimeras. ACS Cent Sci. 2016;2:927–34. 10.1021/acscentsci.6b00280 28058282 PMC5200928

[91] Blackman ML Royzen M Fox JM. Tetrazine ligation: fast bioconjugation based on inverse-electron-demand Diels-Alder reactivity. J Am Chem Soc. 2008;130:13518–9. 10.1021/ja8053805 18798613 PMC2653060

[92] Fischer ES Böhm K Lydeard JR Yang H Stadler MB Cavadini S Structure of the DDB1-CRBN E3 ubiquitin ligase in complex with thalidomide. Nature. 2014;512:49–53. 10.1038/nature13527 25043012 PMC4423819

[93] Raina K Lu J Qian Y Altieri M Gordon D Rossi AMK PROTAC-induced BET protein degradation as a therapy for castration-resistant prostate cancer. Proc Natl Acad Sci U S A. 2016;113:7124–9. 10.1073/pnas.1521738113 27274052 PMC4932933

[94] Bolden JE Tasdemir N Dow LE van Es JH Wilkinson JE Zhao Z Inducible *in vivo* silencing of Brd4 identifies potential toxicities of sustained BET protein inhibition. Cell Rep. 2014;8:1919–29. 10.1016/j.celrep.2014.08.025 25242322 PMC4234106

[95] Velema WA Szymanski W Feringa BL. Photopharmacology: beyond proof of principle. J Am Chem Soc. 2014;136:2178–91. 10.1021/ja413063e 24456115

[96] Liu J Chen H Ma L He Z Wang D Liu Y Light-induced control of protein destruction by opto-PROTAC. Sci Adv. 2020;6:eaay5154. 10.1126/sciadv.aay5154 32128407 PMC7034987

[97] Kounde CS Shchepinova MM Saunders CN Muelbaier M Rackham MD Harling JD A caged E3 ligase ligand for PROTAC-mediated protein degradation with light. Chem Commun (Camb). 2020;56:5532–5. 10.1039/d0cc00523a 32297626

[98] Naro Y Darrah K Deiters A. Optical control of small molecule-induced protein degradation. J Am Chem Soc. 2020;142:2193–7. 10.1021/jacs.9b12718 31927988 PMC7229639

[99] Xue G Wang K Zhou D Zhong H Pan Z. Light-induced protein degradation with photocaged PROTACs. J Am Chem Soc. 2019;141:18370–4. 10.1021/jacs.9b06422 31566962

[100] Pfaff P Samarasinghe KTG Crews CM Carreira EM. Reversible spatiotemporal control of induced protein degradation by bistable photoPROTACs. ACS Cent Sci. 2019;5:1682–90. 10.1021/acscentsci.9b00713 31660436 PMC6813558

[101] Jin YH Lu MC Wang Y Shan WX Wang XY You QD Azo-PROTAC: novel light-controlled small-molecule tool for protein knockdown. J Med Chem. 2020;63:4644–54. 10.1021/acs.jmedchem.9b02058 32153174

[102] Reynders M Matsuura BS Bérouti M Simoneschi D Marzio A Pagano M PHOTACs enable optical control of protein degradation. Sci Adv. 2020;6:eaay5064. 10.1126/sciadv.aay5064 32128406 PMC7034999

[103] Zhao Q Ren C Liu L Chen J Shao Y Sun N Discovery of SIAIS178 as an effective BCR-ABL degrader by recruiting von Hippel-Lindau (VHL) E3 ubiquitin ligase. J Med Chem. 2019;62:9281–98. 10.1021/acs.jmedchem.9b01264 31539241

[104] Zhang X Thummuri D Liu X Hu W Zhang P Khan S Discovery of PROTAC BCL-XL degraders as potent anticancer agents with low on-target platelet toxicity. Eur J Med Chem. 2020;192:112186. 10.1016/j.ejmech.2020.112186 32145645 PMC7433031

[105] Su S Yang Z Gao H Yang H Zhu S An Z Potent and preferential degradation of CDK6 via proteolysis targeting chimera degraders. J Med Chem. 2019;62:7575–82. 10.1021/acs.jmedchem.9b00871 31330105 PMC6790125

[106] Churcher I. Protac-induced protein degradation in drug discovery: breaking the rules or just making new ones? J Med Chem. 2018;61:444–52. 10.1021/acs.jmedchem.7b01272 29144739

[107] Lipinski CA Lombardo F Dominy BW Feeney PJ. Experimental and computational approaches to estimate solubility and permeability in drug discovery and development settings. Adv Drug Deliv Rev. 2001;46:3–26. 10.1016/s0169-409x(00)00129-0 11259830

[108] Neklesa T Snyder LB Willard RR Vitale N Pizzano J Gordon DA ARV-110: an oral androgen receptor PROTAC degrader for prostate cancer. J Clin Oncol. 2019;37:259.

[109] Egbert M Whitty A Keserű GM Vajda S. Why some targets benefit from beyond rule of five drugs. J Med Chem. 2019;62:10005–25. 10.1021/acs.jmedchem.8b01732 31188592 PMC7102492

[110] Shultz MD. Two decades under the influence of the rule of five and the changing properties of approved oral drugs. J Med Chem. 2019;62:1701–14. 10.1021/acs.jmedchem.8b00686 30212196

[111] Veber DF Johnson SR Cheng HY Smith BR Ward KW Kopple KD. Molecular properties that influence the oral bioavailability of drug candidates. J Med Chem. 2002;45:2615–23. 10.1021/jm020017n 12036371

[112] DeGoey DA Chen HJ Cox PB Wendt MD. Beyond the rule of 5: lessons learned from Abbvie’s drugs and compound collection. J Med Chem. 2018;61:2636–51. 10.1021/acs.jmedchem.7b00717 28926247

[113] Doak BC Zheng J Dobritzsch D Kihlberg J. How beyond rule of 5 drugs and clinical candidates bind to their targets. J Med Chem. 2016;59:2312–27. 10.1021/acs.jmedchem.5b01286 26457449

[114] Poongavanam V Doak BC Kihlberg J. Opportunities and guidelines for discovery of orally absorbed drugs in beyond rule of 5 space. Curr Opin Chem Bio. 2018;44:23–9. 10.1016/j.cbpa.2018.05.010 29803972

[115] Edmondson SD Yang B Fallan C. Proteolysis targeting chimeras (PROTACs) in ‘beyond rule-of-five’ chemical space: recent progress and future challenges. Bioorg Med Chem Lett. 2019;29:1555–64. 10.1016/j.bmcl.2019.04.030 31047748

[116] Matthews SJ McCoy C. Thalidomide: a review of approved and investigational uses. Clin Ther. 2003;25:342–95. 10.1016/s0149-2918(03)80085-1 12749503

[117] Webster R Didier E Harris P Siegel N Stadler J Tilbury L PEGylated proteins: evaluation of their safety in the absence of definitive metabolism studies. Drug Metab Dispos. 2007;35:9–16. 10.1124/dmd.106.012419 17020954

[118] Baumann A Tuerck D Prabhu S Dickmann L Sims J. Pharmacokinetics, metabolism and distribution of PEGs and PEGylated proteins: quo vadis? Drug Discov Today. 2014;19:1623–31. 10.1016/j.drudis.2014.06.002 24929223

[119] Webster R Elliott VL Park BK Walker D Hankin M Taupin P. PEG and PEG conjugates toxicity: towards an understanding of the toxicity of PEG and its relevance to PEGylated biologicals. In: Veronese FMeditor. PEGylated Protein Drugs: Basic Science and Clinical Applications. Basel: Birkhäuser; 2009. pp. 127–46.

[120] Cantrill C Chaturvedi P Rynn C Schaffland JP Walter I Wittwer MB. Fundamental aspects of DMPK optimization of targeted protein degraders. Drug Discov Today. 2020;25:969–82. 10.1016/j.drudis.2020.03.012 32298797

[121] Jaime-Figueroa S Buhimschi AD Toure M Hines J Crews CM. Design, synthesis and biological evaluation of proteolysis targeting chimeras (PROTACs) as a BTK degraders with improved pharmacokinetic properties. Bioorg Med Chem Lett. 2020;30:126877. 10.1016/j.bmcl.2019.126877 31879210 PMC7318425

[122] Zeng M Xiong Y Safaee N Nowak RP Donovan KA Yuan CJ Exploring targeted degradation strategy for oncogenic KRAS^G12C^. Cell Chem Biol. 2020;27:19–31.e6. 10.1016/j.chembiol.2019.12.006 31883964

[123] Steinebach C Ng YLD Sosič I Lee CS Chen S Lindner S Systematic exploration of different E3 ubiquitin ligases: an approach towards potent and selective CDK6 degraders. Chem Sci. 2020;11:3474–86. 10.1039/D0SC00167H 33133483 PMC7552917

[124] Shah RR Redmond JM Mihut A Menon M Evans JP Murphy JA Hi-JAK-ing the ubiquitin system: the design and physicochemical optimisation of JAK PROTACs. Bioorg Med Chem. 2020;28:115326. 10.1016/j.bmc.2020.115326 32001089

[125] Mares A Miah AH Smith IED Rackham M Thawani AR Cryan J. Extended pharmacodynamic responses observed upon PROTAC-mediated degradation of RIPK2. Commun Biol. 2020;3:140. 10.1038/s42003-020-0868-6 32198438 PMC7083851

[126] Chessum NEA Sharp SY Caldwell JJ Pasqua AE Wilding B Colombano G Demonstrating in-cell target engagement using a pirin protein degradation probe (CCT367766). J Med Chem. 2018;61:918–33. 10.1021/acs.jmedchem.7b01406 29240418 PMC5815658

[127] Reist M Carrupt PA Francotte E Testa B. Chiral inversion and hydrolysis of thalidomide: mechanisms and catalysis by bases and serum albumin, and chiral stability of teratogenic metabolites. Chem Res Toxicol. 1998;11:1521–8. 10.1021/tx9801817 9860497

[128] Chan KH Zengerle M Testa A Ciulli A. Impact of target warhead and linkage vector on inducing protein degradation: comparison of bromodomain and extra-terminal (BET) degraders derived from triazolodiazepine (JQ1) and tetrahydroquinoline (I-BET726) BET inhibitor scaffolds. J Med Chem. 2018;61:504–13. 10.1021/acs.jmedchem.6b01912 28595007 PMC5788402

[129] Huang HT Dobrovolsky D Paulk J Yang G Weisberg EL Doctor ZM A chemoproteomic approach to query the degradable kinome using a multi-kinase degrader. Cell Chem Biol. 2018;25:88–99.e6. 10.1016/j.chembiol.2017.10.005 29129717 PMC6427047

[130] Zhang X Thummuri D He Y Liu X Zhang P Zhou D Utilizing PROTAC technology to address the on-target platelet toxicity associated with inhibition of BCL-XL. Chem Commun. 2019;55:14765–8. 10.1039/C9CC07217A PMC7057339 31754664

[131] Potjewyd F Turner AMW Beri J Rectenwald JM Norris-Drouin JL Cholensky SH Degradation of polycomb repressive complex 2 with an EED-targeted bivalent chemical degrader. Cell Chem Biol. 2020;27:47–56.e15. 10.1016/j.chembiol.2019.11.006 31831267 PMC7004250

[132] Cheng M Yu X Lu K Xie L Wang L Meng F Discovery of potent and selective epidermal growth factor receptor (EGFR) bifunctional small-molecule degraders. J Med Chem. 2020;63:1216–32. 10.1021/acs.jmedchem.9b01566 31895569 PMC7318554

[133] Maniaci C Hughes SJ Testa A Chen W Lamont DJ Rocha S Homo-PROTACs: bivalent small-molecule dimerizers of the VHL E3 ubiquitin ligase to induce self-degradation. Nat Commun. 2017;8:830. 10.1038/s41467-017-00954-1 29018234 PMC5635026

[134] Tovell H Testa A Zhou H Shpiro N Crafter C Ciull A Design and characterization of SGK3-PROTAC1, an isoform specific SGK3 kinase PROTAC degrader. ACS Chem Biol. 2019;14:2024–34. 10.1021/acschembio.9b00505 31461270 PMC6757289

[135] Douglass EF Miller CJ Sparer G Shapiro H Spiegel DA. A comprehensive mathematical model for three-body binding equilibria. J Am Chem Soc. 2013;135:6092–9. 10.1021/ja311795d 23544844 PMC3717292

[136] Zhang Y Loh C Chen J Mainolfi N. Targeted protein degradation mechanisms. Drug Discov Today Technol. 2019;31:53–60. 10.1016/j.ddtec.2019.01.001 31200860

[137] Maniaci C Ciulli A. Bifunctional chemical probes inducing protein-protein interactions. Curr Opin Chem Biol. 2019;52:145–56. 10.1016/j.cbpa.2019.07.003 31419624

[138] Testa A Hughes SJ Lucas X Wright JE Ciulli A. Structure-based design of a macrocyclic PROTAC. Angew Chem Int Ed Engl. 2020;59:1727–34. 10.1002/anie.201914396 31746102 PMC7004083

[139] Baud MGJ Lin-Shiao E Zengerle M Tallant C Ciulli A. New synthetic routes to triazolo-benzodiazepine analogues: expanding the scope of the bump-and-hole approach for selective bromo and extra-terminal (BET) bromodomain inhibition. J Med Chem. 2016;59:1492–500. 10.1021/acs.jmedchem.5b01135 26367539 PMC4770307

[140] Demizu Y Shibata N Hattori T Ohoka N Motoi H Misawa T Development of BCR-ABL degradation inducers via the conjugation of an imatinib derivative and a cIAP1 ligand. Bioorg Med Chem Lett. 2016;26:4865–9. 10.1016/j.bmcl.2016.09.041 27666635

[141] Winzker M Friese A Koch U Janning P Ziegler S Waldmann H. Development of a PDEδ-targeting PROTACs that impair lipid metabolism. Angew Chem Int Ed Engl. 2020;59:5595–601. 10.1002/anie.201913904 31829492 PMC7154537

[142] Bian J Ren J Li Y Wang J Xu X Feng Y Discovery of Wogonin-based PROTACs against CDK9 and capable of achieving antitumor activity. Bioorg Chem. 2018;81:373–81. 10.1016/j.bioorg.2018.08.028 30196207

[143] Drummond ML Williams CI. *In silico* modeling of PROTAC-mediated ternary complexes: validation and application. J Chem Inf Model. 2019;59:1634–44. 10.1021/acs.jcim.8b00872 30714732

[144] Yang H Lv W He M Deng H Li H Wu W Plasticity in designing PROTACs for selective and potent degradation of HDAC6. Chem Commun (Camb). 2019;55:14848–51. 10.1039/c9cc08509b 31769449

[145] An Z Lv W Su S Wu W Rao Y. Developing potent PROTACs tools for selective degradation of HDAC6 protein. Protein Cell. 2019;10:606–9. 10.1007/s13238-018-0602-z 30603959 PMC6626596

[146] Wang Z He N Guo Z Niu C Song T Guo Y Proteolysis targeting chimeras for the selective degradation of Mcl-1/Bcl-2 derived from nonselective target binding ligands. J Med Chem. 2019;62:8152–63. 10.1021/acs.jmedchem.9b00919 31389699

[147] Nowak RP DeAngelo SL Buckley D He Z Donovan KA An J Plasticity in binding confers selectivity in ligand-induced protein degradation. Nat Chem Biol. 2018;14:706–14. 10.1038/s41589-018-0055-y 29892083 PMC6202246

[148] Imrie F Bradley AR van der Schaar M Deane CM. Deep generative models for 3D linker design. J Chem Inf Model. 2020;60:1983–95. 10.1021/acs.jcim.9b01120 32195587 PMC7189367

[149] Saur M Hartshorn MJ Dong J Reeks J Bunkoczi G Jhoti H Fragment-based drug discovery using cryo-EM. Drug Discov Today. 2020;25:485–90. 10.1016/j.drudis.2019.12.006 31877353

[150] Scapin G Potter CS Carragher B. Cryo-EM for small molecules discovery, design, understanding, and application. Cell Chem Biol. 2018;25:1318–25. 10.1016/j.chembiol.2018.07.006 30100349 PMC6239957

[151] Nakane T Kotecha A Sente A McMullan G Masiulis S Brown PMGE Single-particle cryo-EM at atomic resolution. bioRxiv: 10.1101/2020.05.22.110189v1 [Preprint]. 2020 [cited 2020 Jun 14]: [31 p.]. Available from: https://www.biorxiv.org/content/10.1101/2020.05.22.110189v1 10.1038/s41586-020-2829-0 PMC7611073 33087931

[152] McCoull W Cheung T Anderson E Barton P Burgess J Byth K Development of a novel B-cell lymphoma 6 (BCL6) PROTAC to provide insight into small molecule targeting of BCL6. ACS Chem Biol. 2018;13:3131–41. 10.1021/acschembio.8b00698 30335946

[153] Hughes SJ Ciulli A. Molecular recognition of ternary complexes: a new dimension in the structure-guided design of chemical degraders. Essays Biochem. 2017;61:505–16. 10.1042/EBC20170041 29118097 PMC5869862

[154] Mayer M Meyer B. Group epitope mapping by saturation transfer difference NMR to identify segments of a ligand in direct contact with a protein receptor. J Am Chem Soc. 2001;123:6108–17. 10.1021/ja0100120 11414845

[155] Dias DM Van Molle I Baud MGJ Galdeano C Geraldes CFGC Ciulli A. Is NMR fragment screening fine-tuned to assess druggability of protein-protein interactions? ACS Med Chem Lett. 2014;5:23–8. 10.1021/ml400296c 24436777 PMC3891296

[156] Bai N Kirubakaran P Karanicolas J. Rationalizing PROTAC-mediated ternary complex formation using Rosetta. bioRxiv: 10.1101/2020.05.27.119347 [Preprint]. 2020 [cited 2020 Jun 11]: [43 p.]. Available from: https://www.biorxiv.org/content/10.1101/2020.05.27.119347v1.full.pdf 10.1101/2020.05.27.119347 PMC8866032 33625214

[157] Zaidman D London N. PRosettaC: Rosetta based modeling of PROTAC mediated ternary complexes. bioRxiv: 10.1101/2020.05.27.119354 [Preprint]. 2020 [cited 2020 Jun 11]: [21 p.]. Available from: https://www.biorxiv.org/content/10.1101/2020.05.27.119354v1.full.pdf 10.1021/acs.jcim.0c00589 PMC7592117 32976709

[158] Scott DE Rooney TPC Bayle ED Mirza T Willems HMG Clarke JH Systematic investigation of the permeability of androgen receptor PROTACs. ACS Med Chem Lett. 2020;11:1539–47. 10.1021/acsmedchemlett.0c00194 32832021 PMC7429968

[159] Dong M Babalhavaeji A Samanta S Beharry AA Woolley GA. Red-shifting azobenzene photoswitches for *in vivo* use. Acc Chem Res. 2015;48:2662–70. 10.1021/acs.accounts.5b00270 26415024

[160] Roberts BL Ma ZX Gao A Leisten ED Yin D Xu W Two-stage strategy for development of proteolysis targeting chimeras and its application for estrogen receptor degraders. ACS Chem Biol. 2020;15:1487–96. 10.1021/acschembio.0c00140 32255606

[161] Burslem GM Bondeson DP Crews CM. Scaffold hopping enables direct access to more potent PROTACs with *in vivo* activity. Chem Commun (Camb). 2020;56:6890–2. 10.1039/d0cc02201b 32519703 PMC7404552

[162] Baud MGJ Lin-Shiao E Cardote T Tallant C Pschibul A Chan KH Chemical biology. A bump- and-hole approach to engineer controlled selectivity of BET bromodomain chemical probes. Science. 2014;346:638–41. 10.1126/science.1249830 25323695 PMC4458378

[163] Runcie AC Zengerle M Chan KH Testa A van Beurden L Baud MGJ Optimization of a “bump-and-hole” approach to allele-selective BET bromodomain inhibition. Chem Sci. 2018;9:2452–68. 10.1039/c7sc02536j 29732121 PMC5909127

[164] Moreau K Coen M Zhang AX Pachl F Castaldi MP Dahl G Proteolysis-targeting chimeras in drug development: a safety perspective. Br J Pharmacol. 2020;177:1709–18. 10.1111/bph.15014 32022252 PMC7070175

[165] Pillow TH Adhikari P Blake RA Chen J Del Rosario G Deshmukh G Antibody conjugation of a chimeric BET degrader enables *in vivo* activity. ChemMedChem. 2020;15:17–25. 10.1002/cmdc.201900497 31674143

